# Proteomic and transcriptomic profiling identifies mediators of anchorage-independent growth and roles of inhibitor of differentiation proteins in invasive lobular carcinoma

**DOI:** 10.1038/s41598-020-68141-9

**Published:** 2020-07-13

**Authors:** Nilgun Tasdemir, Kai Ding, Laura Savariau, Kevin M. Levine, Tian Du, Ashuvinee Elangovan, Emily A. Bossart, Adrian V. Lee, Nancy E. Davidson, Steffi Oesterreich

**Affiliations:** 1 Women’s Cancer Research Center, University of Pittsburgh Medical Center (UPMC) Hillman Cancer Center (HCC), Magee-Womens Research Institute, 204 Craft Avenue, Pittsburgh, PA 15213 USA; 2 Department of Pharmacology and Chemical Biology, University of Pittsburgh School of Medicine, Pittsburgh, PA 15213 USA; 3 Integrative Systems Biology Program, University of Pittsburgh, Pittsburgh, PA USA; 4 Department of Human Genetics, University of Pittsburgh Graduate School of Public Health, Pittsburgh, PA 15261 USA; 5 Department of Pathology, University of Pittsburgh School of Medicine, Pittsburgh, PA 15261 USA; 6 School of Medicine, Tsinghua University, Beijing, 100084 China; 7 Molecular Genetics and Developmental Biology Graduate Program, University of Pittsburgh School of Medicine, Pittsburgh, PA 15213 USA; 8 Fred Hutchinson Cancer Center, Seattle, WA 98109 USA; 9 University of Washington, Seattle, WA 98195 USA

**Keywords:** Cell signalling, Breast cancer

## Abstract

Invasive lobular carcinoma (ILC) is a histological subtype of breast cancer with distinct molecular and clinical features from the more common subtype invasive ductal carcinoma (IDC). ILC cells exhibit anchorage-independent growth in ultra-low attachment (ULA) suspension cultures, which is largely attributed to the loss of E-cadherin. In addition to anoikis resistance, herein we show that human ILC cell lines exhibit enhanced cell proliferation in ULA cultures as compared to IDC cells. Proteomic comparison of ILC and IDC cell lines identified induction of PI3K/Akt and p90-RSK pathways specifically in ULA culture in ILC cells. Further transcriptional profiling uncovered unique upregulation of the inhibitors of differentiation family transcription factors *ID1* and *ID3* in ILC ULA culture, the knockdown of which diminished the anchorage-independent growth of ILC cell lines through cell cycle arrest. We find that *ID1* and *ID3* expression is higher in human ILC tumors as compared to IDC, correlated with worse prognosis uniquely in patients with ILC and associated with upregulation of angiogenesis and matrisome-related genes. Altogether, our comprehensive study of anchorage independence in human ILC cell lines provides mechanistic insights and clinical implications for metastatic dissemination of ILC and implicates ID1 and ID3 as novel drivers and therapeutic targets for lobular breast cancer.

## Introduction

Invasive lobular carcinoma (ILC) is one of the major histological subtypes of breast cancer, which accounts for ~ 10–15% of all cases^1^. Compared to the more common subtype invasive ductal carcinoma (IDC), ILC has a number of unique histological, molecular and clinical characteristics. ILC tumors exhibit single-file growth invading the surrounding stroma in a diffuse, linear pattern^2^. This unusual feature is largely attributed to the hallmark genetic loss of *CDH1*, which encodes the adherens junction protein E-cadherin^3–5^. Despite their favorable prognostic and predictive factors such as expression of the estrogen receptor alpha (ER), belonging mainly to the Luminal A (LumA) subtype and a low proliferative index^6^, ILC tumors paradoxically exhibit more frequent long-term recurrences on endocrine therapy than IDC tumors^7,8^. Furthermore, patients with ILC frequently present with metastatic dissemination to unusual anatomical sites such as the peritoneum, ovaries and gastrointestinal tract^1, 7^, clinical features that are not currently well understood. While recent approaches of synthetic lethality with E-cadherin loss have begun to identify therapeutic targets such as ROS1^9^, there is still an urgent need for novel treatment options to improve the clinical outcome of patients with ILC.

Most mammalian cells need continual anchorage of their integrin receptors to the extracellular matrix (ECM) for sustained downstream signaling pathways such as Focal Adhesion Kinase (FAK)^10,11^. As part of their evolution, some cancer cells acquire an ability to grow in the absence of anchorage, which allows cells that have escaped from the site of the primary tumor to survive in the blood stream and subsequently at foreign matrix environments at secondary organs^12,13^. As such, anchorage-independence is believed to be an important contributor to tumor cell dissemination and a surrogate indicator of the ability for distant metastatic colonization^14,15^. A well-studied mechanism of anchorage-independence is anoikis resistance, which is the ability to survive detachment-induced cell death^16,17^. Beyond anoikis resistance, the contribution of other biological processes to anchorage-independence such as cell proliferation and transition through the cell cycle are less well studied.

Anchorage-independence has previously been described in mouse models of lobular cancer, where combined somatic inactivation of p53 and E-cadherin induces ER-negative metastatic ILC through induction of anoikis resistance^18,19^. As more recently shown in cell lines derived from this mouse model, in the ER-negative human ILC cell line IPH-926 and in the ER-positive IDC cell line MCF7, E-cadherin loss drives anoikis resistance by activation of the PI3K/Akt pathway^20,21^. Furthermore, loss of other adherens junction proteins such as p120-catenin (p120) similarly drive the survival of mouse ILC cell lines and primary metastatic human ILC cells through ROCK1-mediated anoikis resistance^22–24^. To complement these findings, we need additional studies of anchorage-independence in ER-positive human ILC cell lines assessing the potential roles of anoikis resistance and cell proliferation.

We have recently published a comprehensive phenotypic characterization of human breast cancer cells in 2D and 3D cultures and reported a remarkably unique ability of ILC cell lines to grow efficiently in ultra-low attachment (ULA) culture as compared to IDC cells^25^, similar to what has been previously described by the Derksen group^18–24^. Given its potential importance in tumor cell dissemination and metastasis^2,12,14,26^, herein we characterized the cellular and molecular mechanisms underlying the anchorage-independence ability of human ILC cells. Using a series of human IDC cell lines for comparison, our work revealed a combined mechanism of anoikis resistance and sustained cell proliferation driving the survival and growth of human ILC cell lines in ULA culture. In addition to assessing the roles of the previously described regulators of anchorage-independence, our proteomic and transcriptional profiling studies uncovered ILC-unique mediators, including the inhibitor of differentiation family proteins ID1 and ID3.

ID1 and ID3 belong to the family of basic-helix-loop-helix (bHLH) transcription factors; however, they lack DNA binding domains^27^. They inhibit differentiation and sustain proliferation of tumor cells by binding to other bHLH transcription factors, ETS proteins and RB in a dominant-negative manner and preventing them from binding to DNA^27,28^. While a few indirect ID1/ID3 targets have been identified, these are mostly associated with proliferation (downstream of RB) and highly context-dependent^27^. ID1 and ID3 have previously been characterized as part of a common murine and human lung metastatic signature in triple negative breast cancer cells^28,29^ and extensively validated as regulators of metastasis^27,28,30^. ID1 and ID3 have also been implicated in anchorage-independent growth in soft agar in small cell lung cancer^31^; however, they have not previously been studied in ILC. Through a series of functional in vitro experiments using cell lines and in silico analyses of patient tumors, herein we have discovered a role for ID1 and ID3 as novel drivers and potential therapeutic targets for ILC.

## Results

### Anoikis resistance and cell proliferation during ILC anchorage-independent growth

We have previously shown that the ER-positive human ILC cell lines MDA-MB-134 (MM134), SUM44PE (SUM44), MDA-MB-330 (MM330) and BCK4 can grow efficiently in ULA suspension culture, as compared to the limited growth of the ER-positive human IDC cell lines MCF7 and T47D, and the ER-negative human IDC cell line MDA-MB-231 (MM231) under the same conditions relative to their 2D growth^25^. As these results were obtained using the CellTiter-Glo luminescent cell viability reagent, here we repeated these experiments with the FluoReporter fluorescent dsDNA assay, the output of which does not rely upon the metabolic activity of cells. Consistent with our previous results, we observed a remarkable ULA growth ability of ILC versus IDC cell lines relative to their 2D growth (Supplementary Fig. S1a,b). Additionally, we showed that the ER-negative, HER2-positive human breast cancer cell line SKBR3^32^ also exhibits limited ULA growth (Supplementary Fig. S1c) and that seeding the ILC and IDC cell lines at lower or higher starting numbers also yields similar results (Supplementary Fig. S1d,e).

**Figure 1:**
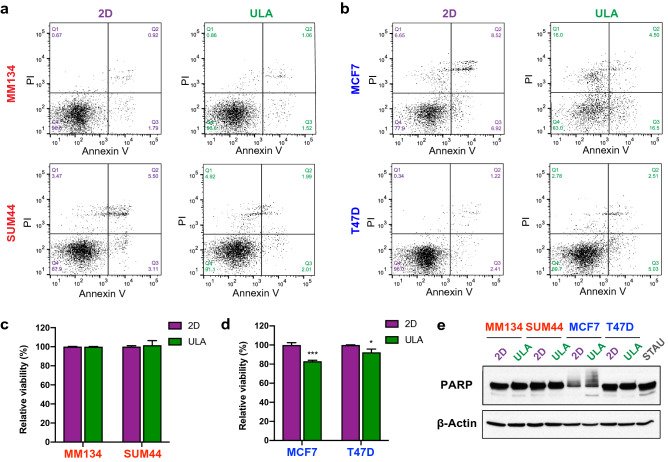
Anoikis resistance of human ILC and IDC cell lines. (**a**,**b**) Representative Annexin V and PI FACS staining plots of the (**a**) ILC (red) cell lines MM134 (top) and SUM44 (bottom) and (**b**) IDC (blue) cell lines MCF7 (top) and T47D (bottom) after 4 days in 2D (left; purple) or ULA (right; green) culture. (**c**,**d**) Quantification of the viable (Q4: Annexin V-/PI-) population in (**c**) ILC and (**d**) IDC cell lines. Data is displayed as mean percentage ± standard deviation relative to the 2D condition in each cell line. Graphs show representative data from two experiments (n = 3). p-values are from *t* tests. *p ≤ 0.05; ***p ≤ 0.001. (**e**) Immunoblotting for PARP in ILC and IDC cell lines after 2 days in 2D or ULA culture. STAU: positive control from T47D cells treated with 1 μM Staurosporine for 5 h. β-Actin was used as a loading control.

Having confirmed the differential growth of ILC and IDC cells in ULA culture, we next assessed the levels of anoikis in these cell types. Annexin V and propidium iodide (PI) flow cytometry (FACS) analysis of MM134, SUM44, MCF7 and T47D cells grown in 2D or ULA showed that while both ILC and IDC cell lines display high levels of anoikis resistance, the effects were stronger in ILC (Fig. 1a,b). Quantification of the double-negative, viable cells indicated no substantial anoikis in MM134 and SUM44, with MCF7 and T47D cells showing ~ 20% and ~ 5% anoikis, respectively (Fig. 1c,d). This anoikis phenotype was further assessed by immunoblotting, which revealed an increase in cleaved PARP (lower band) in ULA versus 2D only in MCF7 cells (Fig. 1e). Furthermore, we confirmed these findings in additional cell lines and observed generally lower levels of anoikis in ILC versus IDC cells (Supplementary Fig. S2).

**Figure 2:**
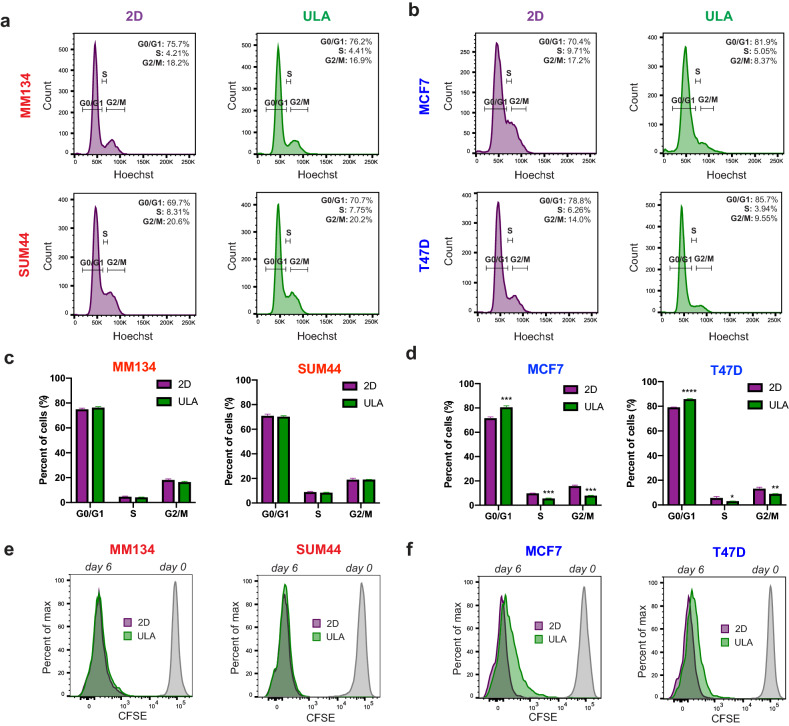
Cell cycle and cell proliferation in ILC and IDC cell lines in 2D and ULA culture. (**a**,**b**) Representative FACS plots from Hoechst staining of the (**a**) ILC (red) cell lines MM134 (top) and SUM44 (bottom) and (**b**) IDC (blue) cell lines MCF7 (top) and T47D (bottom) after 2 days in 2D (left; purple) or ULA (right; green) culture. (**c**,**d**) Quantification of the cells in the indicated phases of the cell cycle based on the gating in (**a**,**b**) in (**c**) ILC and (**d**) IDC cell lines. Data is displayed as mean percentage ± standard deviation (n = 3). p-values are from *t* tests. *p ≤ 0.05; **p ≤ 0.01; ***p ≤ 0.001; ****p ≤ 0.0001. (**e**,**f**) CFSE FACS plots of the (**e**) ILC cell lines MM134 (left) and SUM44 (right) and (**f**) IDC cell lines MCF7 (left) and T47D (right) after initial labeling (day 0; grey) and 6 days in 2D (purple) or ULA (green) culture shown as overlays.

Given the large differences in the viability of ILC and IDC cells in ULA versus 2D conditions (see Supplementary Fig. S1), we reasoned that they might exhibit different levels of proliferation in ULA conditions, in addition to changes in anoikis resistance (see Fig. 1, Supplementary Fig. S2). FACS-based Hoechst staining revealed similar cell cycle profiles for MM134 and SUM44 in 2D and ULA, whereas MCF7 and T47D exhibited more cells arrested in G0/G1, concomitant with a decrease in the percentage of cells in the S and G2/M phases in ULA compared to 2D conditions (Fig. 2a–d). We confirmed these findings by additional FACS analyses, which showed more CFSE-retaining IDC cells in ULA (Fig. 2e,f), as well as lower Ki67 positivity in these cells as compared to 2D (Supplementary Fig. S3), despite similar levels for ILC cells in both conditions and assays. Collectively, these data indicate that the superior relative viability of human ILC cells in ULA conditions versus 2D compared to IDC cells is due to a combined mechanism of anoikis resistance and sustained cell proliferation.

### Roles of known regulators of anchorage-independence in ILC ULA growth

**Figure 3:**
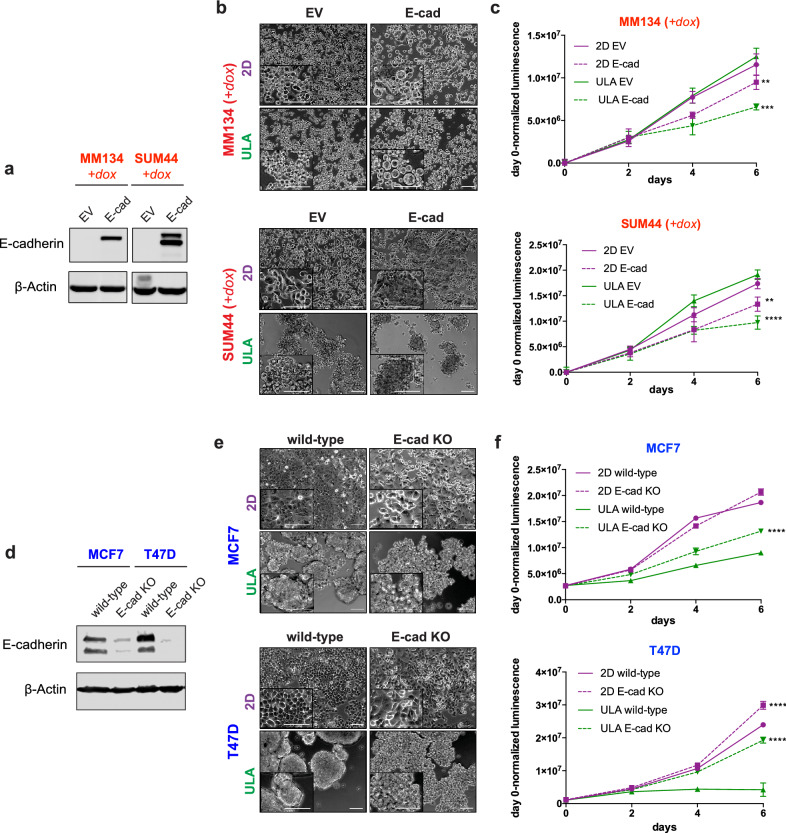
Effects of stable E-cadherin restoration in ILC and knockout in IDC cell lines on cell morphology and viability in 2D and ULA culture. (**a**–**f**) Immunoblotting for E-cadherin (**a**,**d**), morphology (**b**,**e**) and growth (**c**,**f**) in 2D (purple) or ULA (green) culture in the (**a**–**c**) ILC (red) cell lines MM134 (left; top) and SUM44 (right; bottom) stably transfected with a doxycycline (dox)-inducible empty or E-cadherin (E-cad) overexpression vector and (**d**–**f**) IDC (blue) cell lines MCF7 (left; top) and T47D (right; bottom) with CRISPR-mediated stable knockout (KO) of E-cadherin (E-cad). β-Actin was used as a loading control. Scale bar: 100 μm. Graphs show representative data from two–three experiments (n = 6). p-values are from two-way ANOVA comparison of (**c**) empty vector and E-cad or (**f**) wild-type and E-cad KO in 2D and ULA culture separately. **p ≤ 0.01; ***p ≤ 0.001; ****p ≤ 0.0001.

To test the previously described role of E-cadherin in anchorage independence^18,19,33,34^, we stably overexpressed E-cadherin in MM134 and SUM44 cells using a doxycycline-inducible system. Re-introduction of E-cadherin led to tighter cell–cell contacts by morphology and significantly diminished the growth of these ILC cell lines in both 2D and ULA culture, with stronger effects in ULA (Fig. 3a–c, Supplementary Fig. S4). As a complementary approach, we also stably knocked out E-cadherin in MCF7 and T47D cells using CRISPR-mediated genome editing, which led to a rounded cell morphology and partially rescued the growth in ULA culture, but not fully to the levels of growth in 2D culture (Fig. 3d–f). Combined, these data show that E-cadherin regulates the anchorage-independence of ILC and IDC cell lines.

Besides E-cadherin, we assessed the effects of a number of other pathways additionally implicated in anchorage-independence such as YAP/Hippo^35–37^, p120 and Rho/ROCK^22–24,38^. The ROCK inhibitor Y-27632 yielded very similar dose response curves in 2D and ULA cultures (Supplementary Fig. S5a,b) and led to generally elongated morphologies in 2D (Supplementary Fig. S5c,d) for both ILC and IDC cell lines. We noted a small differential effect in MM330 and MCF7 cells only at 10 μM with stronger inhibition in ULA versus 2D and tighter colony formation in ULA. In addition, we also used siRNAs to transiently knockdown ROCK1, p120 and YAP in MM134 and SUM44 cells, where we observed cell line-dependent effects: We failed to observe effects in SUM44 cells, but did note effects of loss of YAP1 and p120 on growth of MM134 cells (Supplementary Fig. S6). For YAP1, the effects were more pronounced in ULA compared to 2D. However, overall the observed effects were relatively minor, and we thus reasoned that there might be additional unique players regulating the growth of human ILC cell lines in ULA.

### Proteomic mediators of ILC anchorage-independent growth

**Figure 4:**
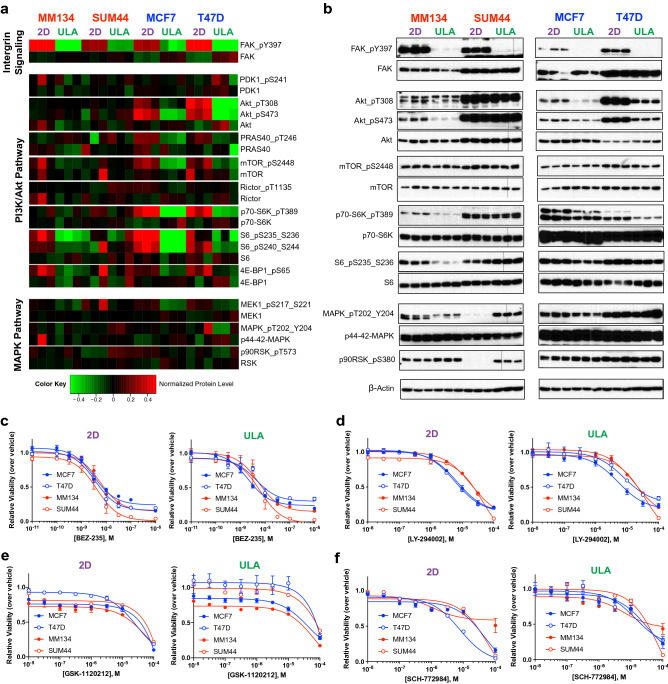
Proteomic profiling and drug treatments of ILC and IDC cell lines in 2D and ULA culture. (**a**,**b**) RPPA (**a**) and Western blot (**b**) analyses of the ILC (red) cell lines MM134 and SUM44 and IDC (blue) cell lines MCF7 and T47D grown in 2D (purple) or ULA (green) culture for 24 h for the indicated pathways and proteins. Three biological replicates are displayed for each condition. β-Actin was used as a loading control. (**c-f**) Dose response curves of the ILC and IDC cell lines from (**a**) treated with the indicated doses of the (**c**) PI3K/mTOR inhibitor BEZ-235, (**d**) PI3K inhibitor LY-294002, (**e**) MEK inhibitor GSK-1120212 and (**f**) MAPK inhibitor SCH-772984 in 2D (purple; left) or ULA (green; right) culture after 4 days.

To uncover potential proteomic mediators of ILC anchorage-independent growth, we next performed Reverse Phase Protein Array (RPPA) assays using extracts from ILC and IDC cell lines after a 24-h culture in 2D or ULA (Fig. 4a, Supplementary Table S1, Supplementary Fig. S7) and confirmed these results by immunoblotting (Fig. 4b). Common to both ILC and IDC cell lines, we observed reduced FAK phosphorylation in ULA versus 2D consistent with inactive integrin signaling in the absence of matrix. Major differences (to be further discussed below) were observed in (i) PI3K/Akt pathway, which was sustained in the ILC ULA cultures (albeit observed at different time points in the two different ILC cell lines), in contrast to its downregulation in IDC in ULA versus 2D, (ii) Ras/MAPK pathway, which was upregulated in the ILC ULA cultures (despite only at the downstream levels and showing uncoupling in MM134 cells), while not changing substantially in IDC in ULA versus 2D.

With regards to the PI3K/Akt pathway, we observed sustained activation in SUM44 cells, which was downregulated in IDC cells in ULA (Fig. 4a,b). Interestingly, while the PDK1-induced phosphorylation of Akt (T308) and the Akt target PRAS40 (T246) were sustained in ULA culture in MM134 cells, the mTOR-induced phosphorylation of Akt (S473) and downstream PI3K/Akt targets were paradoxically downregulated. Given the longer duration of our growth assays, we also assayed the same pathways after 4 days of 2D and ULA culture and observed upregulation in MM134 and downregulation in SUM44 cells in ULA at this time point, suggesting that they were highly dynamically regulated (Supplementary Fig. S8a), consistent with the complexity of the PI3K/Akt pathway^39^.

With regards to the Ras/MAPK pathway, we observed increased phosphorylation of the downstream effector p90RSK in ILC ULA culture, which was not observed in IDC cells (Fig. 4a,b). Interestingly, this phosphorylation was independent of MEK and MAPK activation, as increased p90RSK phosphorylation was observed in MM134 cells in ULA despite decreased upstream signaling. Similarly, T47D cells did not exhibit induction of p90RSK phosphorylation in ULA in the presence of concomitant upstream MAPK signaling. These results suggested an uncoupling of Ras/MAPK activity and downstream p90RSK phosphorylation in ULA conditions.

In follow-up experiments, pharmacological inhibitors targeting PI3K/Akt and Ras/MAPK pathways did not lead to significant shifts in the overall dose response curves between the different cell lines and culture conditions. Of note, the ILC cell lines showed the strongest sensitivity to the PI3K/mTOR dual inhibitor BEZ-235 in ULA at the highest doses; however, similar effects were also observed in 2D (Fig. 4c). Interestingly, LY-294002, which only targets PI3K, had the strongest efficacy in the IDC cell lines in both conditions (Fig. 4d), in agreement with the previously reported sensitizing effects of their activating PI3K mutations^20,40–42^. Treatment with the MEK inhibitor GSK-1120212 showed the most efficacy in MM134 cells in both 2D and ULA (Fig. 4e), consistent with the inactivating *MAP2K4* mutation in this cell line^43^. Conversely, targeting the same pathway further downstream with the MAPK inhibitor SCH-772984 had the least efficacy in this cell line (Fig. 4f), supporting the highly complex and uncoupled regulation of this pathway. Finally, treatment of ILC and IDC cell lines with the p90RSK inhibitor LJH-685 also did not reveal a significant difference between the dose response curves, although full inhibition could not be achieved even at the highest doses (Supplementary Fig. S7b).

### Transcriptomic profiling of ILC anchorage-independent growth and upregulation of *ID1* and *ID3* in ILC ULA culture

**Figure 5:**
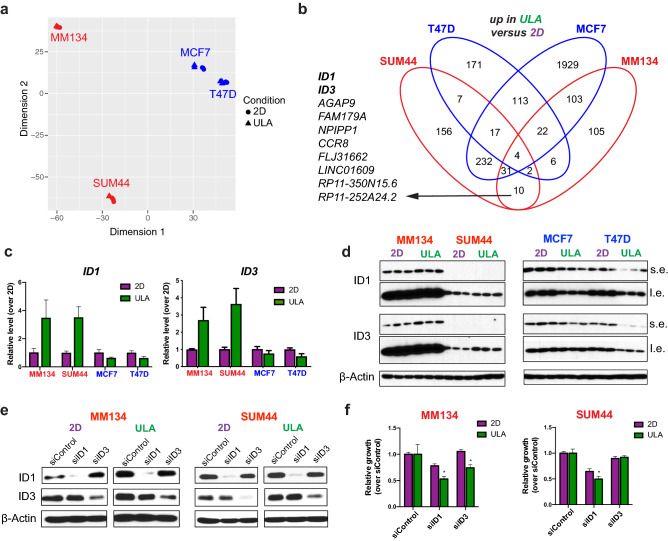
Transcriptomic profiling of ILC and IDC cell lines in 2D and ULA culture and *ID1/ID3* upregulation in ILC ULA culture. (**a**) PCA of transcriptomic data from the ILC (red) and IDC (blue) cell lines grown in 2D (circle) or ULA (triangle) culture for 24 h using the top 5,000 most variable genes ranked by interquartile range. (**b**) Venn diagrams showing the overlap between the genes upregulated in ULA (green) culture as compared to 2D (purple) in the cells from (**a**). The list on the left shows the 10 genes commonly upregulated in the two ILC but not the IDC cell lines. (**c**,**d**) qRT-PCR (**c**) and immunoblotting (**d**) validation of the ID1 and ID3 upregulation in ULA culture in the ILC (left) but not IDC (right) cell lines. Data is displayed as mean ± standard error relative to the 2D condition in each cell line. Graphs show data from three biological replicates. β-Actin was used as a loading control. s.e: short exposure. l.e: long exposure. (**e**,**f**) ID1 and ID3 immunoblotting (**e**) and growth (**f**) of MM134 (left) and SUM44 (right) cell lines 4 days after transient transfection with the indicated siRNAs. Data is displayed as mean ± standard deviation relative to siControl in each condition in each cell line (n = 6). p-values are from *t* tests between 2D and ULA for each siRNA. *p ≤ 0.05.

To complement the proteomic studies above, we next performed RNA-Seq to investigate the transcriptional outputs of the human ILC and IDC cell lines in different culture conditions. To focus on acute transcriptional changes and to mirror the proteomic profiling experiments, we analyzed cells grown in 2D or ULA culture for 24 h. Despite highly similar overall transcriptional profiles as revealed by Principal Component Analysis (PCA) (Fig. 5a), we identified a number of differentially expressed protein-coding and non-coding RNA genes in the two culture conditions (Fig. 5b, Supplementary Fig. S9a, Supplementary Tables S2–S5). To identify potential targetable drivers of the anchorage independence phenotype, we focused on the ULA-upregulated transcripts in both MM134 and SUM44 cells, which were not shared with MCF7 or T47D, including a total of ten genes (Fig. 5b). Given that *ID1* and *ID3* both encode transcription factors from the inhibitors of differentiation family of proteins, which have previously been implicated in metastasis^28,44^, we decided to follow up on these two genes.

**Figure 6:**
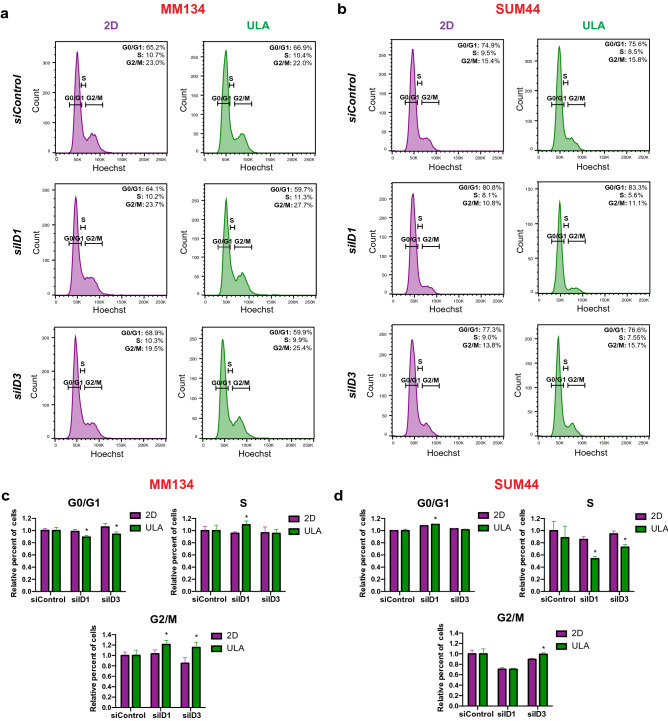
Cell cycle analysis in ILC cell lines with ID1 or ID3 knockdown in 2D and ULA culture. (**a**,**b**) Representative FACS plots from Hoechst staining of the ILC cell lines (**a**) MM134 and (**b**) SUM44 6 days after transient transfection with the indicated siRNAs in 2D (left; purple) or ULA (right; green) culture. (**c**,**d**) Quantification of the cells in the indicated phases of the cell cycle based on the gating in (**a**,**b**) in (**c**) MM134 and (**d**) SUM44 ILC cell lines. Data is displayed as mean percent of cells ± standard deviation relative to siControl in each condition in each cell line (n = 3). p-values are from *t* tests between 2D and ULA for each siRNA. *p ≤ 0.05.

We initially validated the ULA-induction of ID1 and ID3 at the transcript level in ILC cell lines which additionally revealed a reciprocal downregulation in the IDC cells in ULA versus 2D (Fig. 5c), with generally milder changes at the protein level (Fig. 5d, Supplementary Fig. S9b,c). Next we used siRNAs to knockdown ID1 and ID3 in MM134 and SUM44 cells (Fig. 5e, Supplementary Fig. S9d,e). Transient inhibition of ID1 or ID3 resulted in reduced cell growth in both cell lines, generally with stronger effects for ID1 than ID3 and in ULA versus 2D culture (Fig. 5f). Flow cytometry analysis following ID1 and ID3 knockdown revealed a marked decrease in the G0/G1 phase and a concomitant increase in the G2/M phase in MM134 cells (Fig. 6a,c). In contrast, SUM44 cells exhibited a marked increase in the G0/G1 phase and substantial decreases in the S and G2/M phases (Fig. 6b,d). Meanwhile, no substantial effects were seen in apoptosis following ID1 and ID3 knockdown (Supplementary Fig. S10). Taken together, these data implicate ID1 and ID3 as novel drivers of ILC anchorage-independent growth and potential therapeutic targets.

### High *ID1*/*ID3* expression is associated with worse disease specific survival and upregulation of matrisome and angiogenesis-related genes in ILC tumors

**Figure 7:**
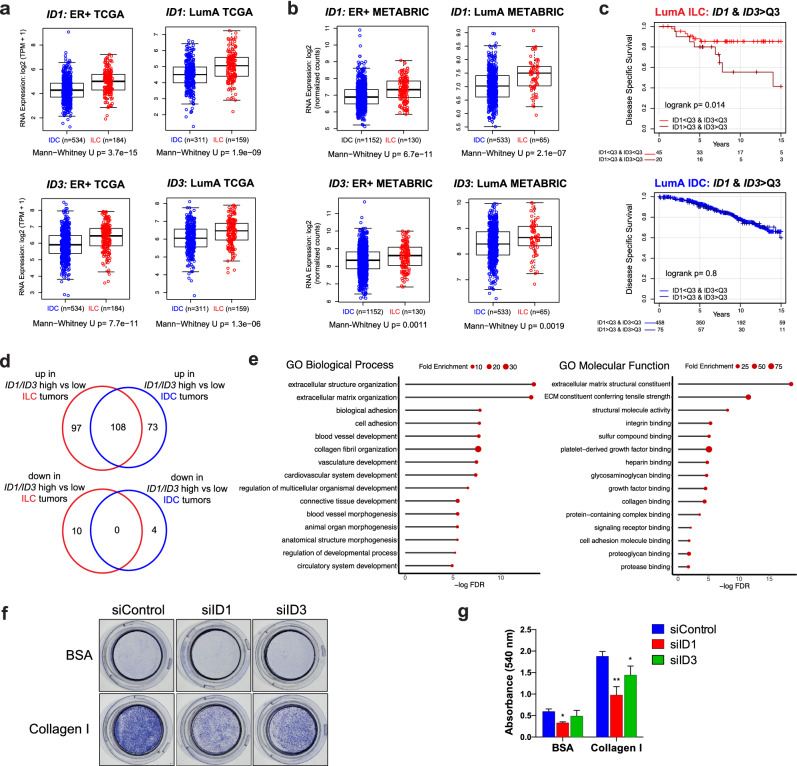
*ID1* and *ID3* expression and function in human breast tumors. (**a**,**b**) mRNA levels of *ID1* (top) and *ID3* (bottom) in ER-positive (left) and LumA (right) ILC (red) and IDC (blue) tumors from the (**a**) TCGA and (**b**) METABRIC cohorts. p-values are from Mann–Whitney *U* test. (**c**) Disease-specific survival curves for combined *ID1* and *ID3* expression in Luminal A ILC (top) and IDC (bottom) patients from the METABRIC cohort divided by third quartile (Q3) levels. p-values are from log-rank test. (**d**) Venn diagrams of genes upregulated (top) and downregulated (bottom) in *ID1/ID3* high versus low tumors in the ILC (red) and IDC (blue) cohorts from the Kaplan Meier plots in (**c**). (**e**) Gene Ontology (GO) Biological Process (left) and Molecular Function (right) terms enriched in the 97 ILC-only upregulated genes from (**d**). (**f**,**g**) Images (**f**) and quantification (**g**) of crystal violet-stained BSA and Collagen I inserts from haptotaxis assays in SUM44 cells transiently transfected with the indicated siRNAs. p-values are form ordinary one-way ANOVA with Dunnett multiple comparison test to siControl. *p ≤ 0.05; **p ≤ 0.01.

To assess the clinical relevance of our findings, we analyzed RNA-Seq data from human breast tumors in The Cancer Genome Atlas (TCGA) collection^6^, which revealed higher *ID1* and *ID3* mRNA expression in both ER-positive and LumA ILC versus IDC tumors (Fig. 7a). Within the molecular subtypes of ILC as defined by the TCGA^6^, we observed lower *ID1* and *ID3* expression in the proliferative subtype as compared to the immune-related and reactive-like subtypes (Supplementary Fig. S11a). Similar results were generally observed in tumors from the METABRIC cohort^45^ (Fig. 7b; Supplementary Fig. S11b), suggesting that ID1 and ID3 may regulate processes other than proliferation in attached ILC tumors, in contrast to their role in anchorage-independence described earlier. Finally, we found that high combined *ID1* and *ID3* expression is correlated with significantly lower disease-specific survival in the LumA ILC but not LumA IDC patients in the METABRIC cohort (Fig. 7c).

In order to gain mechanistic insights into the prognostic significance of *ID1/ID3* expression uniquely in ILC and to decipher ILC-specific *ID1/ID3*-associated potential target genes, we compared the gene expression between *ID1/ID3* high versus low ILC and IDC METABRIC tumors (Fig. 7d, Supplementary Table S7). The genes uniquely downregulated in *ID1/ID3* high versus low ILC tumors (n = 10) were enriched in GO pathways related to the cell cycle (including *CDC20, CENPM, PTTG1, UBE2C)*, further supporting a role beyond proliferation (Supplementary Table S8). The genes uniquely upregulated in *ID1/ID3* high versus low ILC tumors (n = 97) were enriched in GO pathways such as angiogenesis (including *ACKR3*, *ANGPTL4, NRP1, SCG2*), ECM constituents (including 11 genes encoding collagen chains), ECM organization (including the matrix remodeling genes *CTSK, LOX, PLOD2*, *TIMP2*) and cell–matrix adhesion (including the collagen receptor *DDR2, EPDR1, FERMT2, OLFML2B)* (Fig. 7e, Supplementary Table S8).

In follow-up experiments, we functionally validated some of these findings in our ILC cell lines. We had previously shown that ILC cell lines do not exhibit chemotaxis (migration to soluble attractants) to FBS but some of them exhibit haptotaxis (migration towards substrate-bound attractants) to ECM components^25^. Therefore, we transiently knocked down ID1 and ID3 in SUM44 cells and observed a reduced ability of these cells to migrate towards and adhere to Collagen I in haptotaxis experiments using trans-well Boyden chambers (Fig. 7f,g). Combined, these data suggest that ID1 and ID3 may drive the progression of ILC through unique mechanisms in attached versus anchorage-independent conditions and might constitute novel therapeutic targets in ILC, which warrant further investigation.

## Discussion

Invasive lobular carcinoma is a unique histological subtype of breast cancer that exhibits distinct molecular and clinical features from IDC^1^. Our previous work has identified a unique anchorage-independence ability of human ILC cell lines in ULA suspension culture^25^. Herein, we further characterized this interesting phenotype and uncovered a unique, combined mechanism of anoikis resistance and sustained cell proliferation, along with novel mediators of growth in detached culture, which were not shared with IDC cells. While anoikis resistance has been the major focus of existing work on anchorage-independence^14,15,17^, the contribution of cell cycle progression and cell proliferation to this phenotype has been much less studied^16,46–48^. Importantly, we need additional studies on mechanisms of anchorage independence in human ER-positive ILC cell lines.

Given the established role of anchorage-independence in tumor cell dissemination^2,12,14,26^, our findings may have a number of important clinical implications. Although ILC and IDC tumors both exhibit metastases, ILC tumors are associated with more frequent late recurrences. From this perspective, the superior ability of ILC cells to survive in detached conditions might allow them to persist at low levels of proliferation in foreign matrix conditions for extended periods of time. Furthermore, another unique feature of ILC tumors is their colonization of unique anatomical sites. It will be important to further study the matrix compositions of different metastatic organs and assess whether the ILC-specific sites are less permissive for re-establishing cell-ECM contacts, which would favor the survival of detached ILC versus IDC cells given our in vitro data and provide insights into metastatic organotropism.

Our molecular profiling experiments revealed few ULA-induced genes and pathways that were common to both ILC cell lines and not shared with IDC cells. The heterogeneity in the ULA-triggered transcriptional and proteomic changes, as well as in the timing of signaling activation, between MM134 and SUM44 cells suggests that these two cell types might potentially represent different ILC subtypes. This hypothesis is further supported by their highly separated clustering in our PCA analysis and harboring of different mutations^25,43,49^, which might allow convergent but non-overlapping mechanisms of adaptation to detached growth. Although the 24-h time point of suspension culture had previously been successfully utilized to uncover transcripts induced by detachment in triple negative breast cancer^50^, the highly dynamic signaling we observed in ILC justifies further profiling studies focusing on earlier and later time points. In addition, as the RPPA only covers ~ 220 total and phosphoproteins, more comprehensive assays such as mass spectrometry coupled with a complex systems biology approach are more likely to help fully understand the multi-dimensional regulation of signaling pathways unique to ILC suspension culture.

Although we detected ILC-unique ULA-induced PI3K/Akt activation, we did not observe significant shifts in the dose response curves to a PI3K/mTOR dual inhibitor. These data are in agreement with a recent study showing similar dose responses in adherent and suspension culture to Akt inhibitors in ER-negative mouse and human ILC cell lines, as well as reporting PI3K/Akt activation in the ER-positive IDC cell line MCF7 following knockout of E-cadherin^20^. While we noted stronger effects of the PI3K/mTOR dual inhibitor in ILC versus IDC cells in ULA only at the highest doses, a single PI3K inhibitor was conversely more effective in IDC versus ILC cell lines in both culture conditions despite decreased PI3K/Akt signaling in ULA, likely due to the PI3K mutations in MCF7 and T47D^20,40–42^. Furthermore, we observed ILC-unique ULA-induced phosphorylation of p90RSK; however, a p90RSK inhibitor did not reveal any significantly differential sensitivity between the cell types and culture conditions. Given that full inhibition could not be achieved even at the highest doses of this compound, this finding warrants further investigation with more potent inhibitors. Interestingly, p90RSK has previously been implicated in soft agar and Matrigel growth of MM134 and mouse ILC cell lines downstream of FGFR1 and MEK/MAPK^51^. Here we report that ULA culture induces ILC-unique p90RSK phosphorylation, which is uncoupled from upstream MEK/MAPK signaling in the *MAP2K4* and *K-RAS* mutant MM134 cells^43,49^.

Our RNA-Seq profiling identified ILC-unique ULA-induction of transcripts encoding the inhibitor of differentiation family of proteins ID1 and ID3, which we found to be reciprocally downregulated in IDC ULA culture. In our functional studies, transient siRNA-mediated knockdown of *ID1* or *ID3* in ILC cells resulted in reduced viability and impacted the cell cycle but not apoptosis, effects that were generally stronger for *ID1* than for *ID3* and in ULA versus 2D. This dual effect in both culture conditions suggests that ID1 and ID3 inhibition may be effective on both the attached growth in primary tumors, as well as on the detached growth of disseminated cancer cells. Additionally, these data are consistent with previously reported roles of ID1 and ID3 in sustaining the proliferation of cancer cells^27,28^ via regulation of the G0/G1^52,53^ or G2/M^54,55^ phases of the cell cycle in different contexts. Interestingly, despite the mechanistic differences between the anchorage-independence of mouse and human ILC cell lines uncovered here, a previous study in mouse ILC cell lines reported upregulation of *Id2* in detached culture^23^, suggestive of a common role for the inhibitor of differentiation family proteins in anchorage independence and convergent evolution in these two species.

Through our in silico analysis of ER-positive and LumA tumors from TCGA and METABRIC collections, we discovered significantly higher *ID1* and *ID3* transcript levels in ILC versus IDC, as well as generally lower expression in the proliferative subtype of ILC. Furthermore, combined high expression of *ID1* and *ID3* was correlated with significantly worse disease-specific survival in the ILC but not IDC LumA METABRIC cohorts. High *ID1/ID3* expression in ILC tumors was associated with downregulation of cell cycle-related genes, which is in contrast to the proliferative effects of ID1 and ID3 in anchorage-independent conditions, and with upregulation of pathways associated with angiogenesis and the matrisome. While these results are based on correlation analyses, they are largely consistent with previous reports in other contexts^27,31,44,56^ and should be validated in further functional studies. Taken together, these data suggest that ID1 and ID3 may play roles in multiple stages of tumor growth and metastasis by regulating different processes in attached versus detached cancer cells.

Collectively, our discovery of ID1 and ID3 as novel drivers of ILC disease progression implicate these factors as potential therapeutic targets. Given the difficulty of blocking protein–protein interactions with bHLH transcription factors, initial inhibitors targeting ID1 and ID3, such as the ID1-degrader C527^57^ and Cannabidiol^58^, were highly non-specific. While peptide-conjugated antisense oligonucleotides have allowed more precise targeting, they have not been very amenable to clinical translation^59^. The recently discovered small molecule AGX51, which inhibits ID proteins by targeting them for ubiquitin-mediated proteolysis, has shown strong anti-tumor effects, good toleration and lack of acquired resistance in murine models of colorectal and lung cancer^60^. Further clinical development of this promising first-in-class compound, as well as discovery of novel ID inhibitors, will likely yield agents that can be combined with endocrine therapy and potentially improve the clinical outcomes of patients with invasive lobular breast cancer.

## Methods

### Cell culture

MDA-MB-134-VI (MM134), MDA-MB-330 (MM330), MCF7, T47D, MDA-MB-231 (MM231) and SKBR3 cells were obtained from the American Type Culture Collection. SUM44PE (SUM44) cells were purchased from Asterand and BCK4 cells were kindly provided by Britta Jacobsen, University of Colorado Anschutz, CO. Cell lines were maintained as previously described^25^ in the following media (Life Technologies) with 10% FBS: MM134 and MM330 in 1:1 DMEM:L-15, MCF7 and MM231 in DMEM, T47D in RPMI, SKBR3 in McCoy’s 5A, BCK4 in MEM with non-essential amino acids (Life Technologies) and insulin (Sigma-Aldrich). SUM44 cells were maintained as described^61^ in DMEM-F12 with 2% charcoal stripped serum and supplements. Cell lines were routinely tested to be mycoplasma free using MycoAlert Mycoplasma Detection Kit (Lonza; #LT07-418), authenticated by the University of Arizona Genetics Core by Short Tandem Repeat DNA profiling and kept in continuous culture for < 6 months.

### Transient transfection, siRNAs, plasmids, and drugs

ON-TARGETplus SMARTpool small interfering RNAs (siRNAs) were purchased from Dharmacon: ID1 (#L-005051-00-0005), ID3 (#L-009905-00-0005), ROCK1 (#L-003536-00-0005), p120 (#L-012572-00-0005), YAP (#L-012200-00-0005), non-targeting control (#D-001810-10-50). Cells were reverse-transfected with 1 pmol/10 nM of each siRNA in 96-well plates using Lipofectamine RNAiMAX (Life Technologies) and Opti-MEM (Thermo Fisher Scientific) following manufacturer’s instructions. pCDNA3 backbone was from Invitrogen and hE-cadherin-pcDNA3 was a gift from Barry Gumbiner (Addgene plasmid # 45769). Plasmids were forward transfected into cells using FuGENE 6 (Promega). BEZ-235, LY-294002, GSK-1120212, SCH-772984 and LJH-685 were purchased from Selleck Chemicals. Y-27632 was purchased from Sigma Aldrich.

### E-cadherin overexpression and CRISPR knockout

Human E-cadherin was subcloned by cutting from pCDNA3-E-cadherin with XbaI and EcoRV and ligating into the entry vector pENTR1A digested with XmnI and XbaI. pENTR1A-E-cadherin was then used in Gateway cloning (Thermo Fisher Scientific) to generate the destination vector pINDUCER20-E-cadherin. Lentiviruses were generated and cells were transduced as previously described^62^. Stably transduced cells were selected using 1 mg/ml Geneticin (Thermo Fisher Scientific). Cells were plated the day before addition of 1 μg/ml doxycycline (Sigma Aldrich). Knockout of E-cadherin was performed as previously described^63^ by utilizing the Gene Knockout Kit (V1) from Synthego (Redwood City, California) and cells were used as pools.

### Cell viability and anchorage-independence assays

2D and ULA growth assays were performed as previously described^25^. ILC (15,000/96-well; 300,000/6-well) and IDC (5,000/96-well; 100,000/6-well) cells were seeded in regular (Thermo Fisher Scientific) or ULA (Corning Life Sciences) 96-well plates. Due to the smaller size and slower proliferation of ILC cell lines compared to IDC cells, these cell numbers were determined empirically to yield optimal, log-phase growth of the cells in 2D and to yield similar confluences at assay end points. Where indicated, results were also verified by plating the cells at different cell densities. Cells were assayed using CellTiter-Glo (Promega), FluoReporter Blue Fluorometric dsDNA Quantitation Kit (Invitrogen) or PrestoBlue Cell Viability Reagent (Thermo Fisher Scientific). Data was captured on a Promega GloMax or Perkin Elmer plate reader.

### Cell proliferation, cell cycle, apoptosis and haptotaxis assays

Cells were seeded at 300,000/well (ILC) and 100,000/well (IDC) in 6-well 2D and ULA plates in triplicates. For proliferation assays, cells were stained with 0.01 μM carboxyfluorescein succinimidyl ester (CFSE; Thermo Fisher Scientific) for 20 min at room temperature in serum-free media on day 0. At the indicated time points, cells were harvested by trypsinization of 2D cultures in plates and ULA cultures in tubes for 5 min at 37 °C, followed by neutralization with serum-containing media and washing with PBS. Ki67/7-AAD staining was performed using the FITC Mouse Anti-Ki67 set (#556026; BD Biosciences) following manufacturer recommendations and gating on cells stained with an isotype control antibody. For cell cycle analysis, cells were stained with Hoechst (Thermo Fisher Scientific) at 20 mg/ml for 30 min at 37 °C and then briefly with Propidium Iodide (BD Biosciences) to gate on viable cells. For apoptosis assays, cells were stained with APC-Annexin V (BD Biosciences; #550474) and PI in 1X Annexin binding buffer for 15 min at room temperature. Samples were acquired on an LSR II Flow cytometer (BD Biosciences) and analyzed using BD FACSDiva and FlowJo softwares (BD Biosciences). Haptotaxis experiments were performed as previously described using 8 μm inserts coated on the underside with a thin layer of Collagen I (ECM582; EMD Millipore)^25^. After overnight serum starvation, 500,000 cells were plated in each insert in serum free media and the amount of migration through the membranes after 72 h was quantified using crystal violet staining.

### Immunoblotting and reverse phase protein arrays (RPPA)

Immunoblots were performed as previously described^25^ using 5% milk powder for blocking and developed using ECL (Sigma-Aldrich). Details of the antibodies used are in Supplementary Table S6. Blots were quantified using Image J software. Uncropped blots are provided in Supplementary Figs. S12–S14. RPPA was performed as previously described^61^. Samples were collected in MD Anderson RPPA lysis buffer and assessed at the Functional Proteomics Core of MD Anderson. Rawlog2 RPPA data is included in Supplementary Table S1. Normalized log2 median-centered values were used to generate heat maps.

### RNA extraction, quantitative PCR and RNA-sequencing (RNA-Seq)

RNA extraction and qRT-PCRs were done as previously described^25^. Primer sequences are included in Supplementary Table S6. RNA-Seq was performed as previously described^64^ using NextSeq 500. Raw sequence data were mapped to hg38 genome (ensembl release version 82) and gene counts were quantified with Salmon (version 0.8.2)^65^ using default settings. Differentially expressed (DE) analysis for the cell line data was performed with the R package DESeq2^66^ and SAM for METABRIC tumors using the following criteria: absolute log2(fold change) > log2(1.5) and Benjamini–Hochberg adjusted p-value < 0.05. The complete list of DE genes for the cell line data is available in Supplementary Tables S3–S6. Venn diagrams were generated using the online Intervene tool^67^. Gene Ontology (GO) pathway analyses were performed using the GO Enrichment Analysis tool at https://geneontology.org and plotted using R Studio.

### Survival analyses

Survival analyses were performed using the METABRIC dataset^45^ as previously described^68^, using data downloaded from the Synapse software platform (syn1688369; Sage Bionetworks, Seattle, WA, USA).

### Statistical analysis

Data analysis was performed using GraphPad Prism. Data is presented as mean ± standard deviation or standard error of means as indicated. Statistical tests used for each figure are indicated in the respective figure legends.

## Supplementary information

Supplementary information

Supplementary Table S1

Supplementary Table S2

Supplementary Table S3

Supplementary Table S4

Supplementary Table S5

Supplementary Table S6

Supplementary Table S7

Supplementary Table S8

## Data Availability

The datasets supporting the conclusions of this article are included within the article (Supplementary Tables S1 to S8 and Supplementary Figures S1 to S14). RNA-seq data is available at the GEO database (GSE130650).
