# Supplementary material for: Proteomic and transcriptomic profiling identifies mediators of anchorage-independent growth and roles of inhibitor of differentiation proteins in invasive lobular carcinoma
Source: Sci Rep. 2020 Jul 13;10:11487. doi: 10.1038/s41598-020-68141-9 (PMC7359337; doi:10.1038/s41598-020-68141-9)
Supplement: Supplementary file 1 — Supplementary information [file 41598_2020_68141_MOESM1_ESM.pdf]

Supplementary Data for:

Proteomic and Transcriptomic Profiling Identifies Mediators of Anchorage-Independent Growth and Roles of Inhibitor of Differentiation Proteins in Invasive Lobular Carcinoma

Nilgun Tasdemir[1,2], Kai Ding[1,3], Laura Savariau[1,4], Kevin M. Levine[1,5], Tian Du[1,6], Ashuvinee Elangovan[1,7], Emily A. Bossart[1,2], Adrian V. Lee[1,2], Nancy E. Davidson[8,9], Steffi Oesterreich[1,2,*]

[1]Women's Cancer Research Center, University of Pittsburgh Medical Center (UPMC) Hillman Cancer Center (HCC), Magee-Womens Research Institute, Pittsburgh, PA 15213, USA
[2]Department of Pharmacology & Chemical Biology, University of Pittsburgh School of Medicine, Pittsburgh, PA 15213, USA
[3]Integrative Systems Biology Program, University of Pittsburgh , Pittsburgh, PA, USA
[4]Department of Human Genetics, University of Pittsburgh Graduate School of Public Health, Pittsburgh, PA 15261, USA
[5]Department of Pathology, University of Pittsburgh School of Medicine, PA 15261, USA
[6]School of Medicine, Tsinghua University, Beijing 100084, China
[7]Molecular Genetics and Developmental Biology Graduate Program, University of Pittsburgh School of Medicine, Pittsburgh, PA 15213, USA.
[8]Fred Hutchinson Cancer Center, Seattle, WA 98109, USA
[9]University of Washington, Seattle WA 98195, USA

[*]**Corresponding Author:**
Steffi Oesterreich, PhD
oesterreichs@upmc.edu
Phone: 4126418555
Women's Cancer Research Center
UPMC Hillman Cancer Center
204 Craft Avenue
Pittsburgh, PA 15213, USA

Supplementary Table and Supplementary Figure Legends

Supplementary Table S1. Raw log2 RPPA data.

Supplementary Table S2. Differentially regulated genes in ULA versus 2D in MM134 cells.

Supplementary Table S3. Differentially regulated genes in ULA versus 2D in SUM44 cells.

Supplementary Table S4. Differentially regulated genes in ULA versus 2D in MCF7 cells.

Supplementary Table S5. Differentially regulated genes in ULA versus 2D in T47D cells.

Supplementary Table S6. List of antibodies and primers used.

Supplementary Table S7. List of differentially regulated genes in *ID1/ID3* high versus low ILC and IDC tumors from the METABRIC cohort.

Supplementary Table S8. GO enrichment analysis for genes differentially regulated in *ID1/ID3* high versus low ILC and IDC tumors from the METABRIC cohort.

Supplementary Figure S1. Growth of ILC and IDC cell lines in 2D and ULA culture. (**a-b**) Relative growth over day 0 of the (**a**) ILC (red) cell lines MM134 (top) and SUM44 (bottom) and (**b**) IDC (blue) cell lines MCF7 (top) and T47D (bottom) plated at the indicated cell numbers in 2D (purple) or ULA (green) culture. Values are from FluoReporter Blue dsDNA assay (n=3). (**c-e**) Relative growth over day 0 of (**c**) SKBR3 (**d**) MM134 (top) and SUM44 (bottom) (**e**) MCF7 (top) and T47D (bottom) plated at the indicated cell numbers in 2D or ULA culture.

Values are from CellTiter-Glo assay (n=6). p-values are from two-way ANOVA comparison of 2D and ULA. * $p \leq 0.05$; ** $p \leq 0.01$; *** $p \leq 0.001$; **** $p \leq 0.0001$.

Supplementary Figure S2. Anoikis resistance of additional ILC and IDC cell lines. (**a-b**) Annexin V and PI FACS staining plots of (**a**) MM330 (top; red) and BCK4 (bottom; red) and (**b**) MM231 (top; blue) and SKBR3 (bottom; blue) cells after 4 days in 2D (left; purple) or ULA (right; green) culture. (**c-d**) Quantification of the viable (Q4: Annexin V-/PI-) population in (**c**) ILC and (**d**) IDC cell lines. Data is displayed as relative to the 2D condition in each cell line. (**e**) Immunoblotting for PARP in ILC and IDC cell lines after 2 days in 2D or ULA culture. STAU: positive control from MM231 cells treated with 1 μM Staurosporine for 5 hours. β-Actin was used as a loading control.

Supplementary Figure S3. Ki67 levels in ILC and IDC cell lines in 2D and ULA culture as a read-out of cell proliferation. (**a-b**) Representative FACS plots from Ki67 staining of the (**a**) ILC (red) cell lines MM134 (top) and SUM44 (bottom) and (**b**) IDC (blue) cell lines MCF7 (top) and T47D (bottom) after 4 days in 2D (left; purple) or ULA (right; green) culture. Gates were placed based on isotype staining in each cell line in each condition. (**c-d**) Quantification of the Ki67+ cells based on the gating in (**a-b**) in (**c**) ILC and (**d**) IDC cell lines. Data is displayed as mean percentage +/- standard deviation (n=3). p-values are from t-tests. * $p \leq 0.05$; ** $p \leq 0.01$.

Supplementary Figure S4. Effects of stable E-cadherin restoration in ILC on cell line viability in 2D and ULA culture in the absence of doxycycline. (**a-b**) Immunoblotting for E-cadherin (**a**) and cell viability (**b**) in 2D (purple) or ULA (green) culture in the ILC cell lines MM134 (left; top) and SUM44 (right; bottom) stably transfected with a doxycycline (dox)-inducible empty or

E-cadherin (E-cad) overexpression vector and not treated with dox. β-Actin was used as a loading control. Graphs show representative data from two-three experiments (n=6).

Supplementary Figure S5. Effects of ROCK inhibition on the viability and morphology of ILC and IDC cell lines in 2D and ULA culture. (**a-b**) Dose response curves of the (**a**) ILC (red) cell lines MM134 and MM330 (left) and SUM44 and BCK4 (right) and (**b**) IDC (blue) cell lines MCF7 and MM231 (left) and T47D and SKBR3 (right) treated with the indicated doses of the Y-27632 ROCK inhibitor in 2D (purple) or ULA (green) culture after 4 days. Arrows indicate the dose used for the morphology pictures in (**c-d**). (**c-d**) Morphologies of the (**c**) ILC and (**d**) IDC cell lines from (**a-b**) treated with vehicle or 10 µM (10-5 M) Y-27632 for 4 days in 2D or ULA culture. Insets show higher magnification images. Scale bar: 100 µm.

Supplementary Figure S6. Effects of ROCK, p120 and YAP knockdown on the viability of ILC cell lines in 2D and ULA culture. (a-b) Immunoblotting for p120, ROCK1 and YAP (a) and relative growth over day 0 in 2D or ULA culture (b) in the ILC cell lines MM134 (left; top) and SUM44 (right; bottom) transiently transfected with a control, ROCK1, p120 or YAP siRNA. β-Actin was used as a loading control. Graphs show mean +/- standard deviation (n=6). p-values are from two-way ANOVA comparison of 2D and ULA. * $p \leq 0.05$.

Supplementary Figure S7. Full RPPA heat map. Heat map showing the levels of all profiled proteins and phosphoproteins in the RPPA analysis for MM134, SUM44, MCF7 and T47D cell in 2D and ULA culture for 24 hours in biological triplicates.

Supplementary Figure S8. Additional proteomic analysis and drug treatments of ILC and IDC cell lines in 2D and ULA culture. (**a**) Western blot analysis of the ILC (red) cell lines MM134 and SUM44 grown in 2D (purple) or ULA (green) culture for 4 days for the indicated pathways proteins. Three biological replicates are displayed for each condition. β-Actin was used as a loading control. (**b**) Dose response curves of the ILC (red) cell lines MM134 and SUM44 and IDC (blue) cell lines MCF7 and T47D treated with the indicated doses of the p90-RSK inhibitor LJH-685 in 2D (purple; top) or ULA (green; bottom) culture for 4 days.

Supplementary Figure S9. Transcriptomic profiling of ILC and IDC cell lines in 2D and ULA culture. (**a**) Venn diagrams showing the overlap between the genes downregulated after 24 hours in ULA (green) culture as compared to 2D (purple) in ILC (red) and IDC (blue) cell lines. The list on the right shows the 24 genes commonly downregulated in the two ILC but not the IDC cell lines, mostly made up of small nuclear, nucleolar and spliceosomal RNAs. (**b-c**) qRT-PCR validation of the ID1 (left) and ID3 (right) knockdown in MM134 (**b**) and SUM44 (**c**) cells 4 days after transient transfection with the indicated siRNAs. Data is displayed as mean +/- error relative to siControl in each condition in each cell line.

Supplementary Figure S10. Anoikis resistance in ILC cell lines with ID1 or ID3 knockdown in 2D and ULA culture. (**a-b**) Representative Annexin V and PI FACS staining plots of the of the ILC cell lines (**a**) MM134 and (**b**) SUM44 6 days after transient transfection with the indicated siRNAs in 2D (left; purple) or ULA (right; green) culture. (**c-d**) Quantification of the viable (Q4: Annexin V-/PI-) population in (**c**) MM134 and (**d**) SUM44 ILC cell lines from (**a-b**). Data is

displayed as mean percentage +/- standard deviation relative to siControl in each condition in each cell line. Graphs show representative data from two experiments (n=3).

Supplementary Figure S11. ID1 and ID3 expression in the molecular subtypes of ILC. (a-b) mRNA levels of ID1 (top) and ID3 (bottom) in ER-positive (left) and LumA (right) of ILC tumors from the (a) TCGA and (b) METABRIC cohorts according to the the immune-related (red), proliferative (green) and reactive-like (black) molecular subtypes. p-values are from on-way ANOVA with Benjamini-Hochberg multiple comparison test.

Supplementary Figure S12. Uncropped blots related to Figures 1e, 3a, 3d, 4b and Supplementary Figure S4a.

Supplementary Figure S13. Uncropped blots related to Figures 5d and 5e. l.e.: long exposure. s.e.: short exposure.

Supplementary Figure S14. Uncropped blots related to Supplementary Figures S2e, S6a and S8a.

Supplementary Figure S1

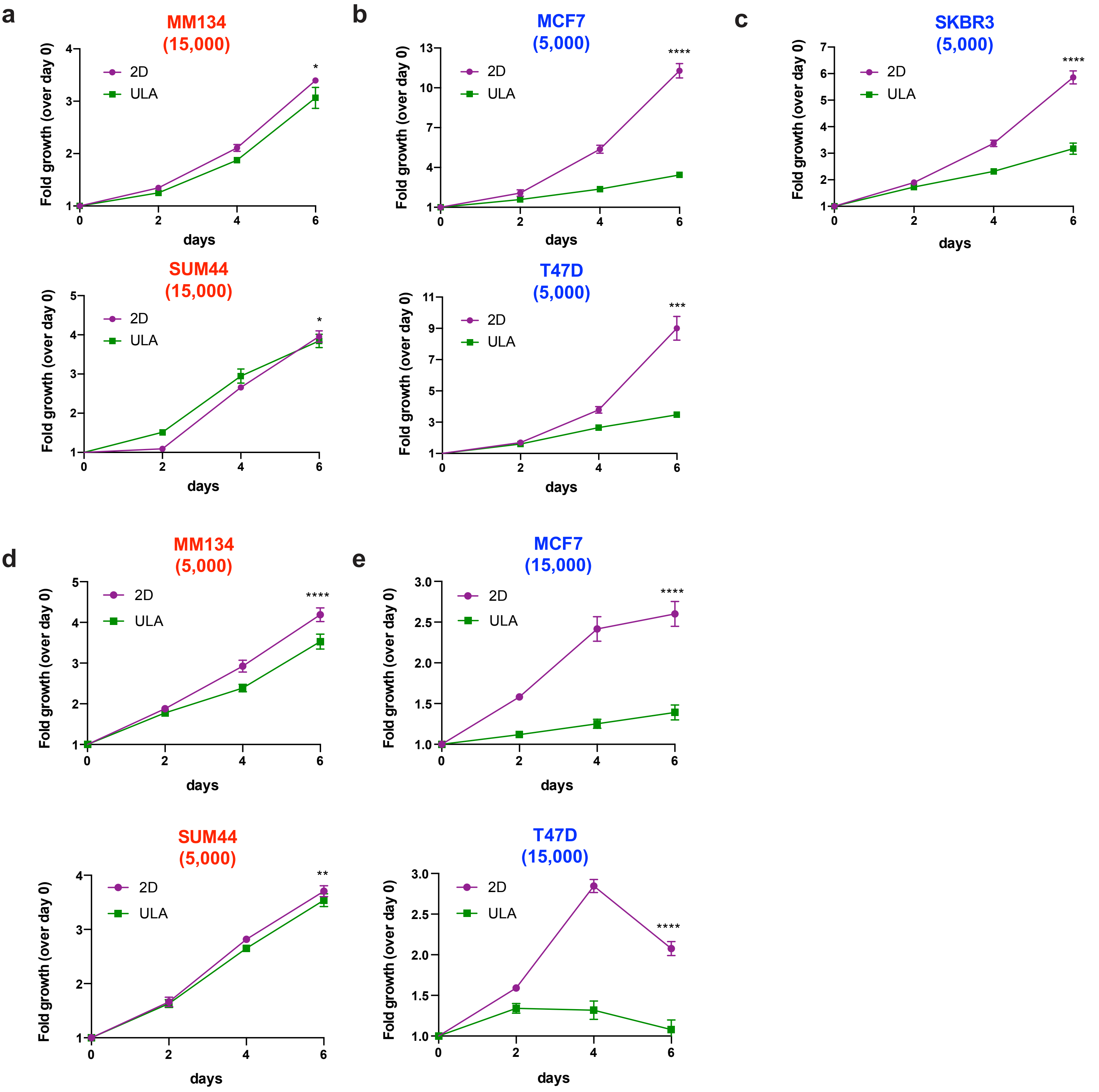

Supplementary Figure S1. Growth of ILC and IDC cell lines in 2D and ULA culture. (**a-b**) Relative growth over day 0 of the (**a**) ILC (red) cell lines MM134 (top) and SUM44 (bottom) and (**b**) IDC (blue) cell lines MCF7 (top) and T47D (bottom) plated at the indicated cell numbers in 2D (purple) or ULA (green) culture. Values are from Fluoreporter Blue dsDNA assay (n=3). (**c-e**) Relative growth over day 0 of (**c**) SKBR3 (**d**) MM134 (top) and SUM44 (bottom) (**e**) MCF7 (top) and T47D (bottom) plated at the indicated cell numbers in 2D or ULA culture. Values are from CellTiter-Glo assay (n=6). p-values are from two-way ANOVA comparison of 2D and ULA. * $p \leq 0.05$; ** $p \leq 0.01$; *** $p \leq 0.001$; **** $p \leq 0.0001$.

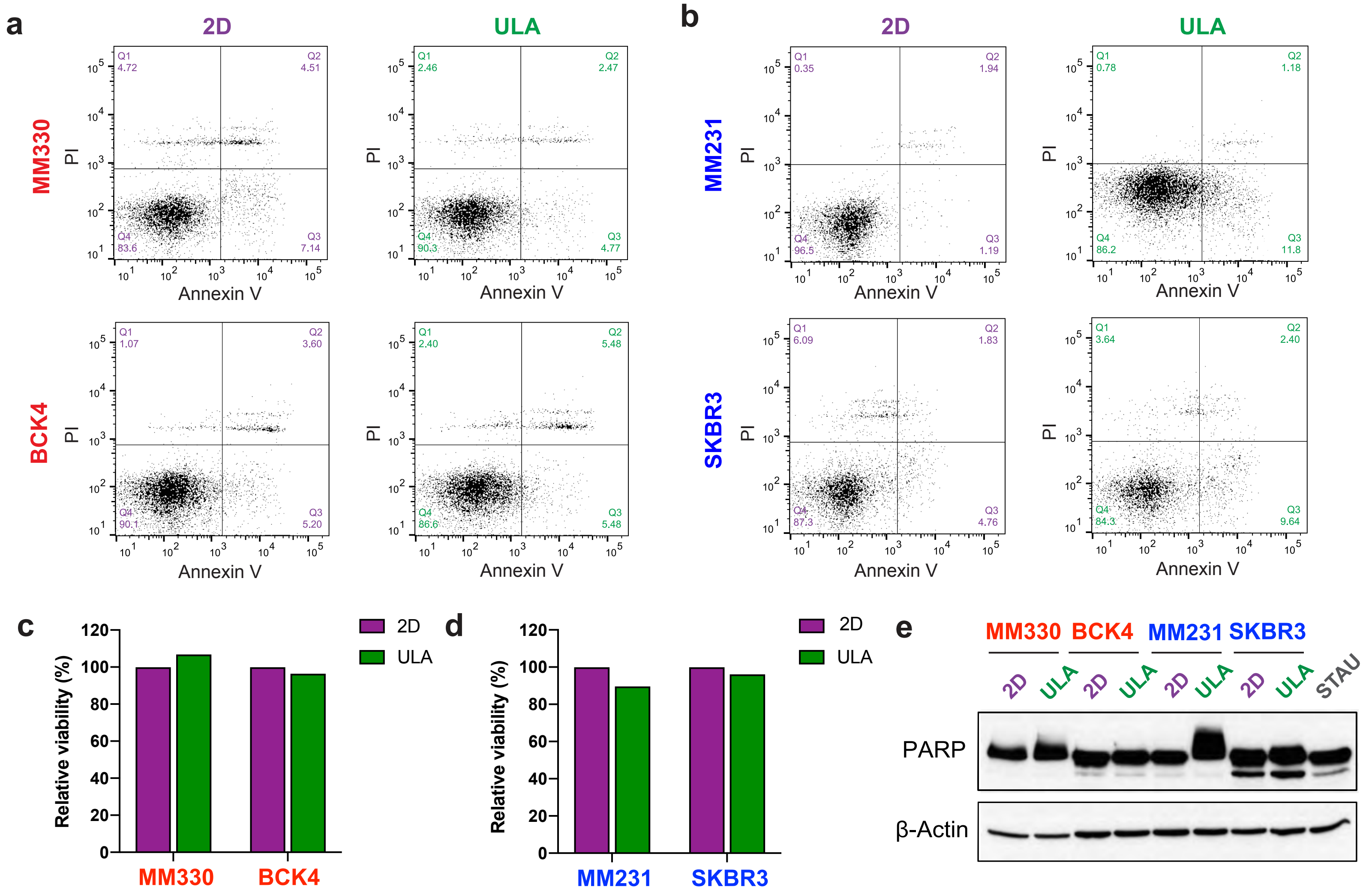

Supplementary Figure S2. Anoikis resistance of additional ILC and IDC cell lines. (**a-b**) Annexin V and PI FACS staining plots of (**a**) MM330 (top; red) and BCK4 (bottom; red) and (**b**) MM231 (top; blue) and SKBR3 (bottom; blue) cells after 4 days in 2D (left; purple) or ULA (right; green) culture. (**c-d**) Quantification of the viable (Q4: Annexin V-/PI-) population in (**c**) ILC and (**d**) IDC cell lines. Data is displayed as relative to the 2D condition in each cell line. (**e**) Immunoblotting for PARP in ILC and IDC cell lines after 2 days in 2D or ULA culture. STAU: positive control from MM231 cells treated with 1 μM Staurosporine for 5 hours. β-Actin was used as a loading control.

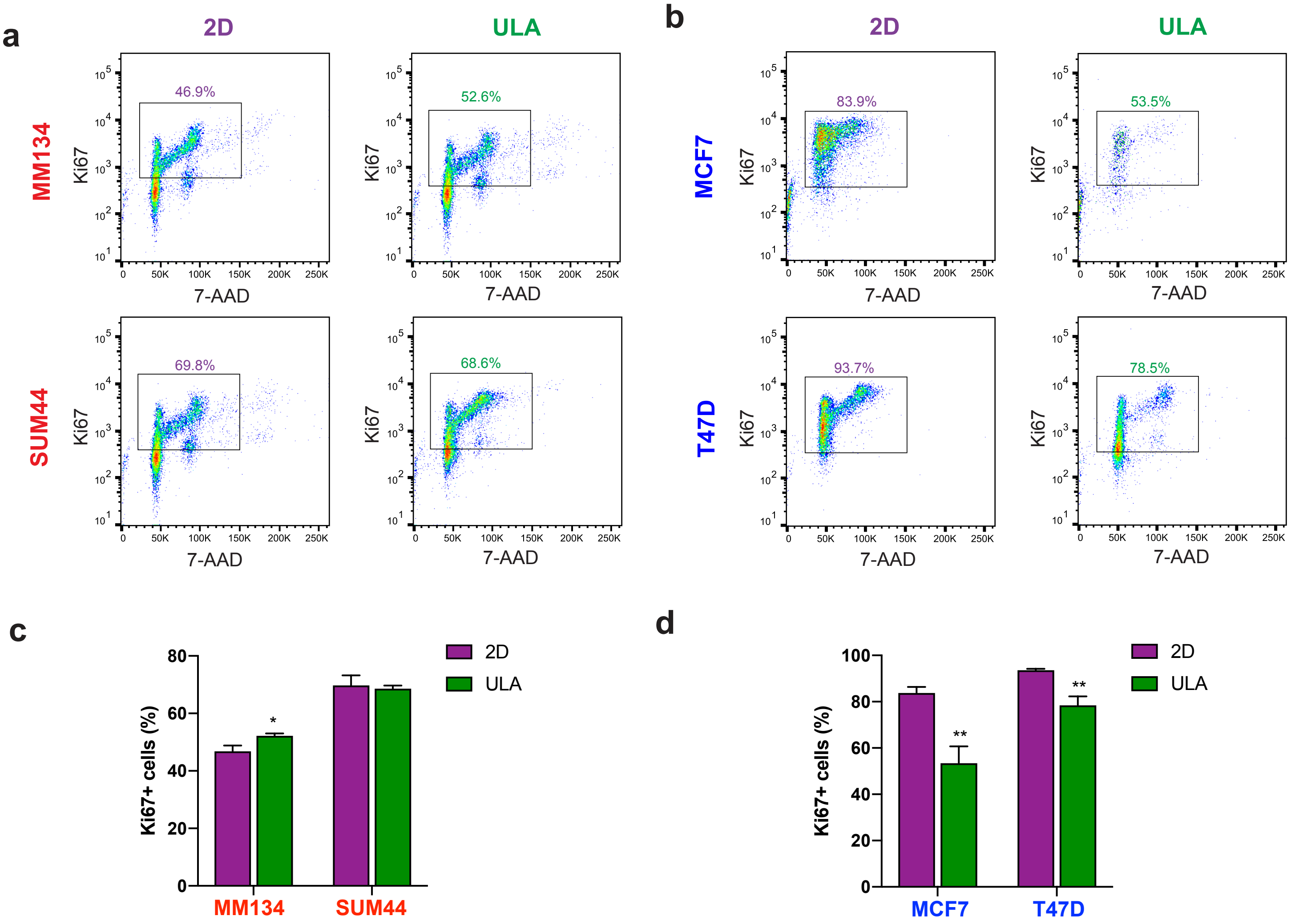

Supplementary Figure S3. Ki67 levels in ILC and IDC cell lines in 2D and ULA culture as a read-out of cell proliferation. (**a-b**) Representative FACS plots from Ki67 staining of the (**a**) ILC (red) cell lines MM134 (top) and SUM44 (bottom) and (**b**) IDC (blue) cell lines MCF7 (top) and T47D (bottom) after 4 days in 2D (left; purple) or ULA (right; green) culture. Gates were placed based on isotype staining in each cell line in each condition. (**c-d**) Quantification of the Ki67+ cells based on the gating in (**a-b**) in (**c**) ILC and (**d**) IDC cell lines. Data is displayed as mean percentage +/- standard deviation (n=3). p-values are from t-tests. * $p \leq 0.05$; ** $p \leq 0.01$

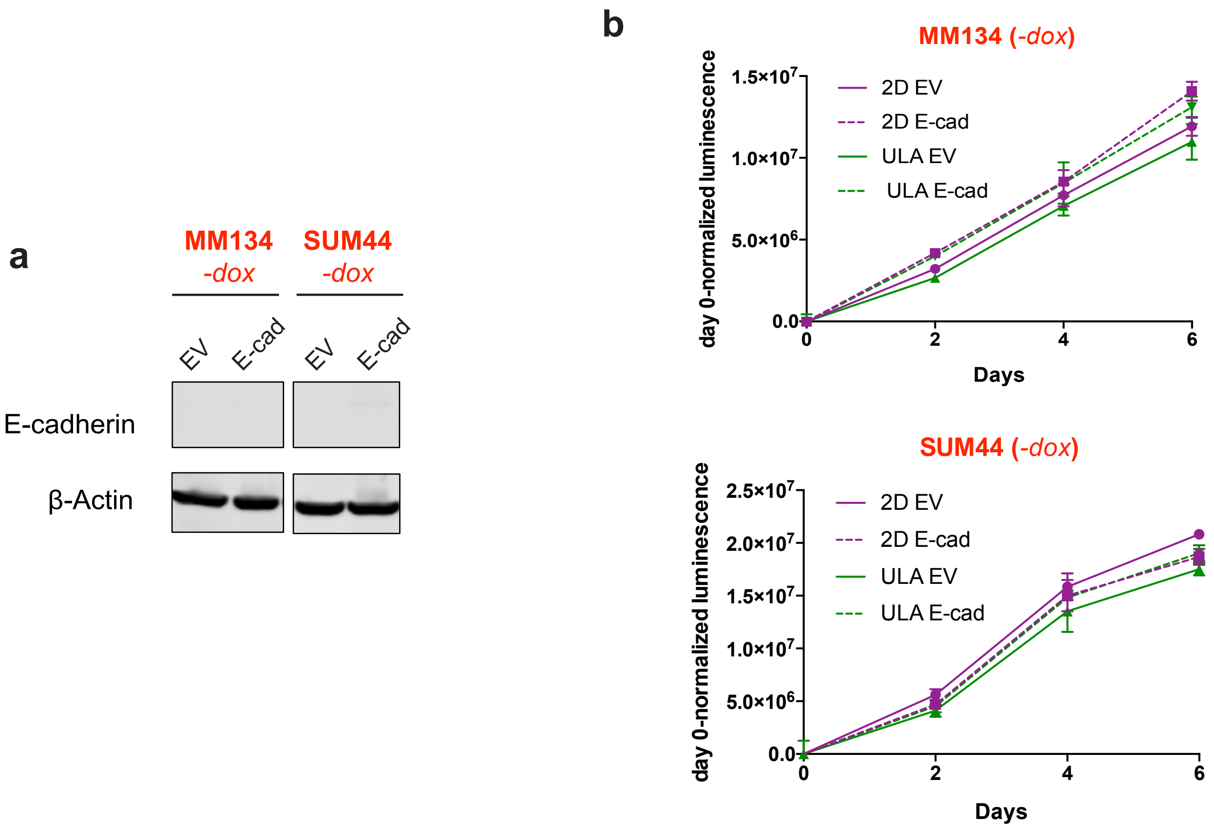

Supplementary Figure S4. Effects of stable E-cadherin restoration in ILC on cell line viability in 2D and ULA culture in the absence of doxycycline. (**a-b**) Immunoblotting for E-cadherin (**a**) and cell viability (**b**) in 2D (purple) or ULA (green) culture in the ILC cell lines MM134 (left; top) and SUM44 (right; bottom) stably transfected with a doxycycline (dox)-inducible empty or E-cadherin (E-cad) overexpression vector and not treated with dox. β-Actin was used as a loading control. Graphs show representative data from two-three experiments (n=6).

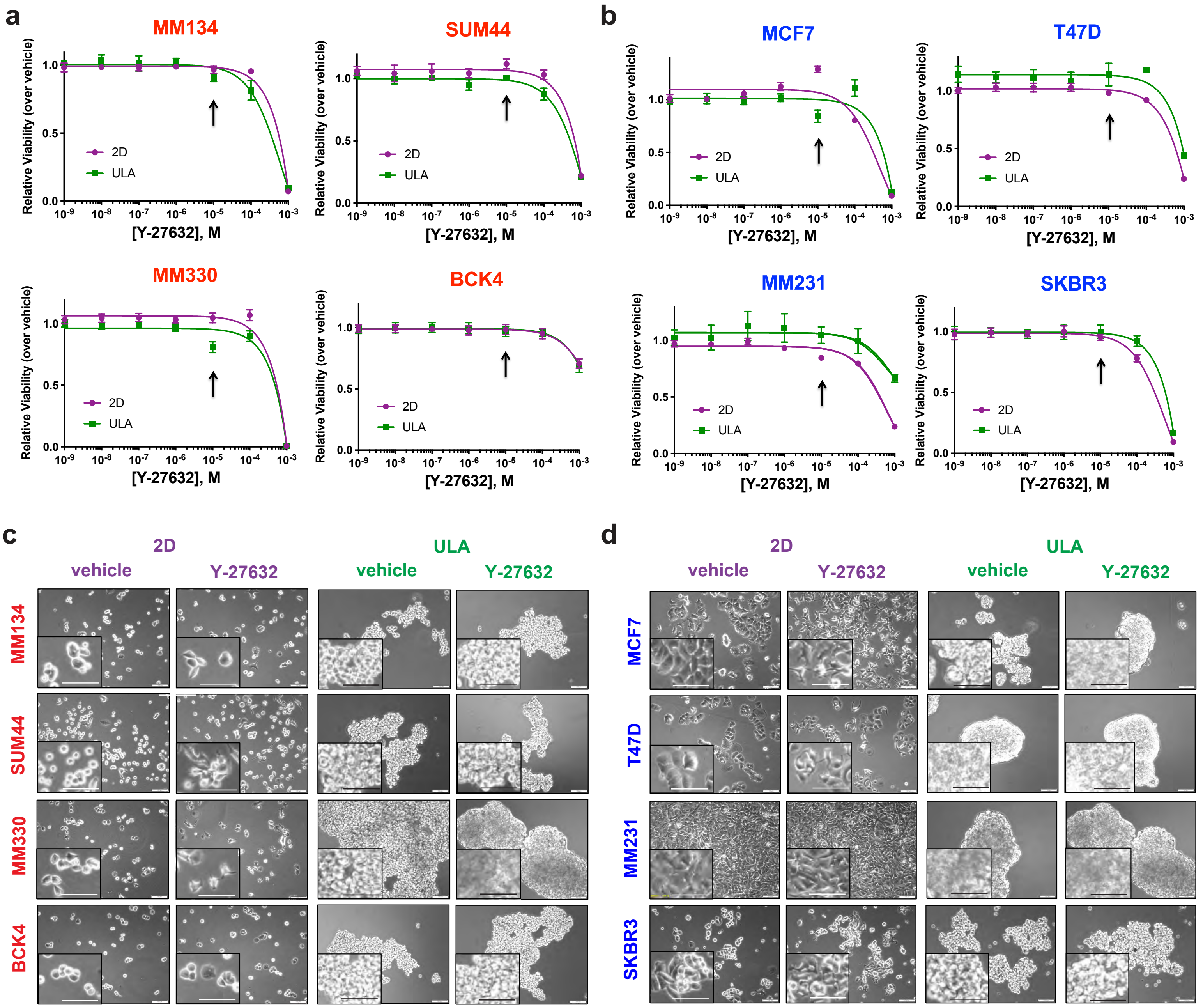

Supplementary Figure S5. Effects of ROCK inhibition on the viability and morphology of ILC and IDC cell lines in 2D and ULA culture. (**a-b**) Dose response curves of the (**a**) ILC (red) cell lines MM134 and MM330 (left) and SUM44 and BCK4 (right) and (**b**) IDC (blue) cell lines MCF7 and MM231 (left) and T47D and SKBR3 (right) treated with the indicated doses of the Y-27632 ROCK inhibitor in 2D (purple) or ULA (green) culture after 4 days. Arrows indicate the dose used for the morphology pictures in (**c-d**). (**c-d**) Morphologies of the (**c**) ILC and (**d**) IDC cell lines from (**a-b**) treated with vehicle or 10 µM (10^{-5} M) Y-27632 for 4 days in 2D or ULA culture. Insets show higher magnification images. Scale bar: 100 µm.

Supplementary Figure S6

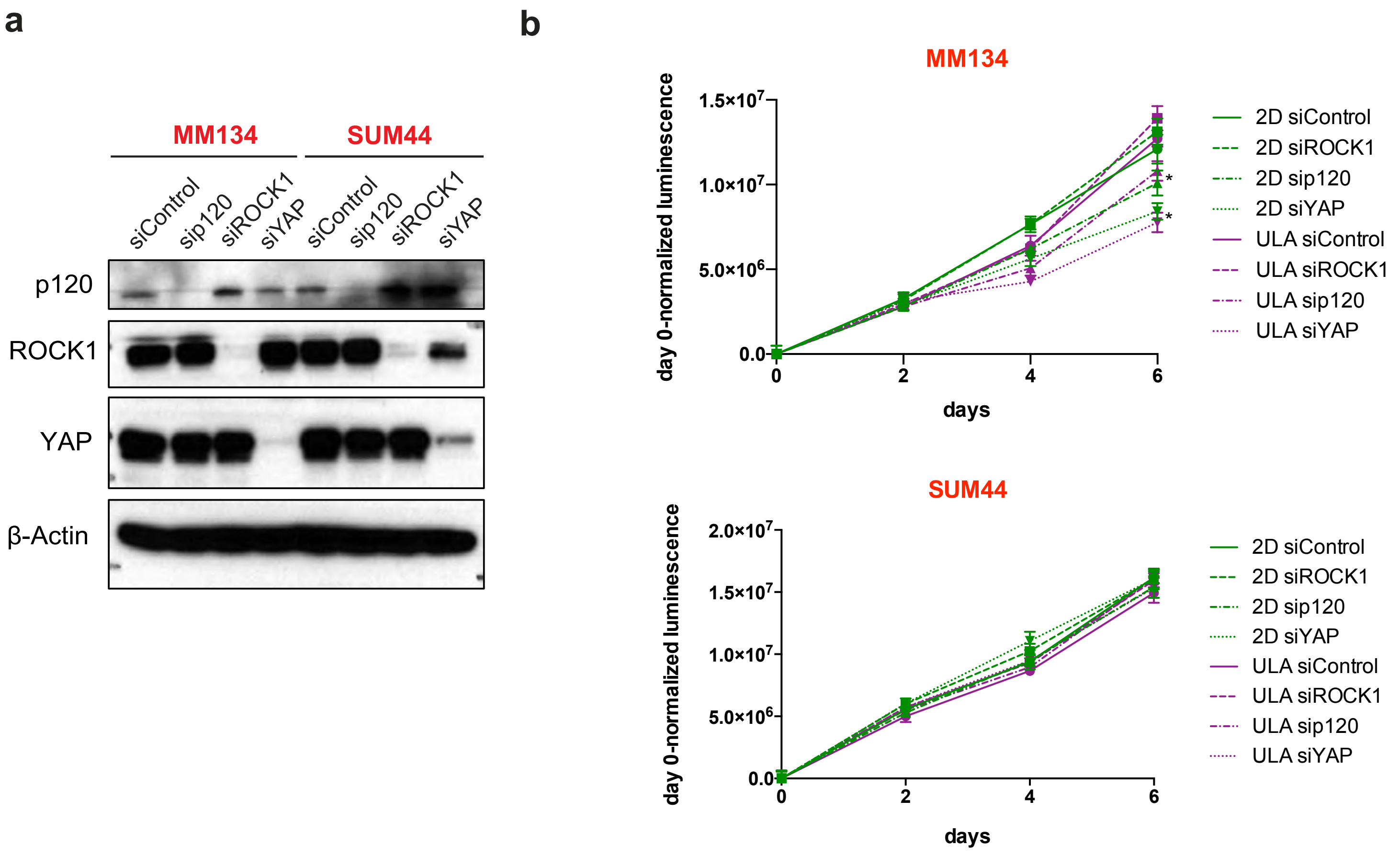

Supplementary Figure S6. Effects of ROCK, p120 and YAP knockdown on the viability of ILC cell lines in 2D and ULA culture. (**a-b**) Immunoblotting for p120, ROCK1 and YAP (**a**) and relative growth over day 0 in 2D or ULA culture (**b**) in the ILC cell lines MM134 (left; top) and SUM44 (right; bottom) transiently transfected with a control, ROCK1, p120 or YAP siRNA. β-Actin was used as a loading control. Graphs show mean +/- standard deviation (n=6). p-values are from two-way ANOVA comparison of 2D and ULA. * $p \leq 0.05$.

Supplementary Figure S7

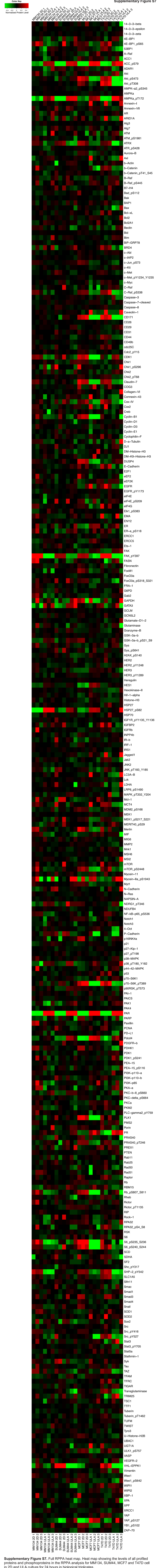

Supplementary Figure S7. Full RPPA heat map. Heat map showing the levels of all profiled proteins and phosphoproteins in the RPPA analysis for MM134, SUM44, MCF7 and T47D cell in 2D and ULA culture for 24 hours in biological triplicates.

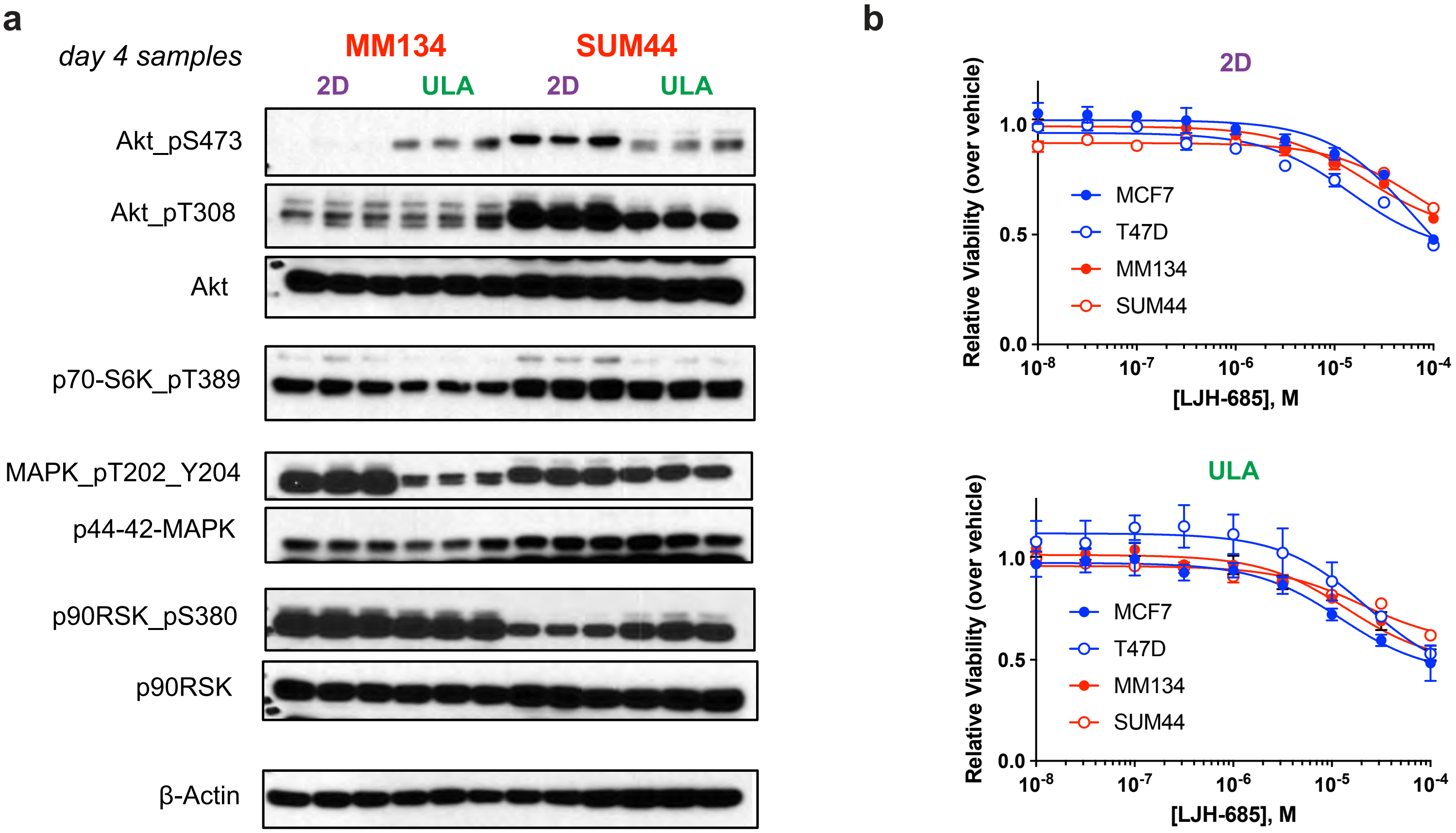

Supplementary Figure S8. Additional proteomic analysis and drug treatments of ILC and IDC cell lines in 2D and ULA culture. (**a**) Western blot analysis of the ILC (red) cell lines MM134 and SUM44 grown in 2D (purple) or ULA (green) culture for 4 days for the indicated pathways proteins. Three biological replicates are displayed for each condition. β-Actin was used as a loading control. (**b**) Dose response curves of the ILC (red) cell lines MM134 and SUM44 and IDC (blue) cell lines MCF7 and T47D treated with the indicated doses of the p90-RSK inhibitor LJH-685 in 2D (purple; top) or ULA (green; bottom) culture for 4 days.

Supplementary Figure S9

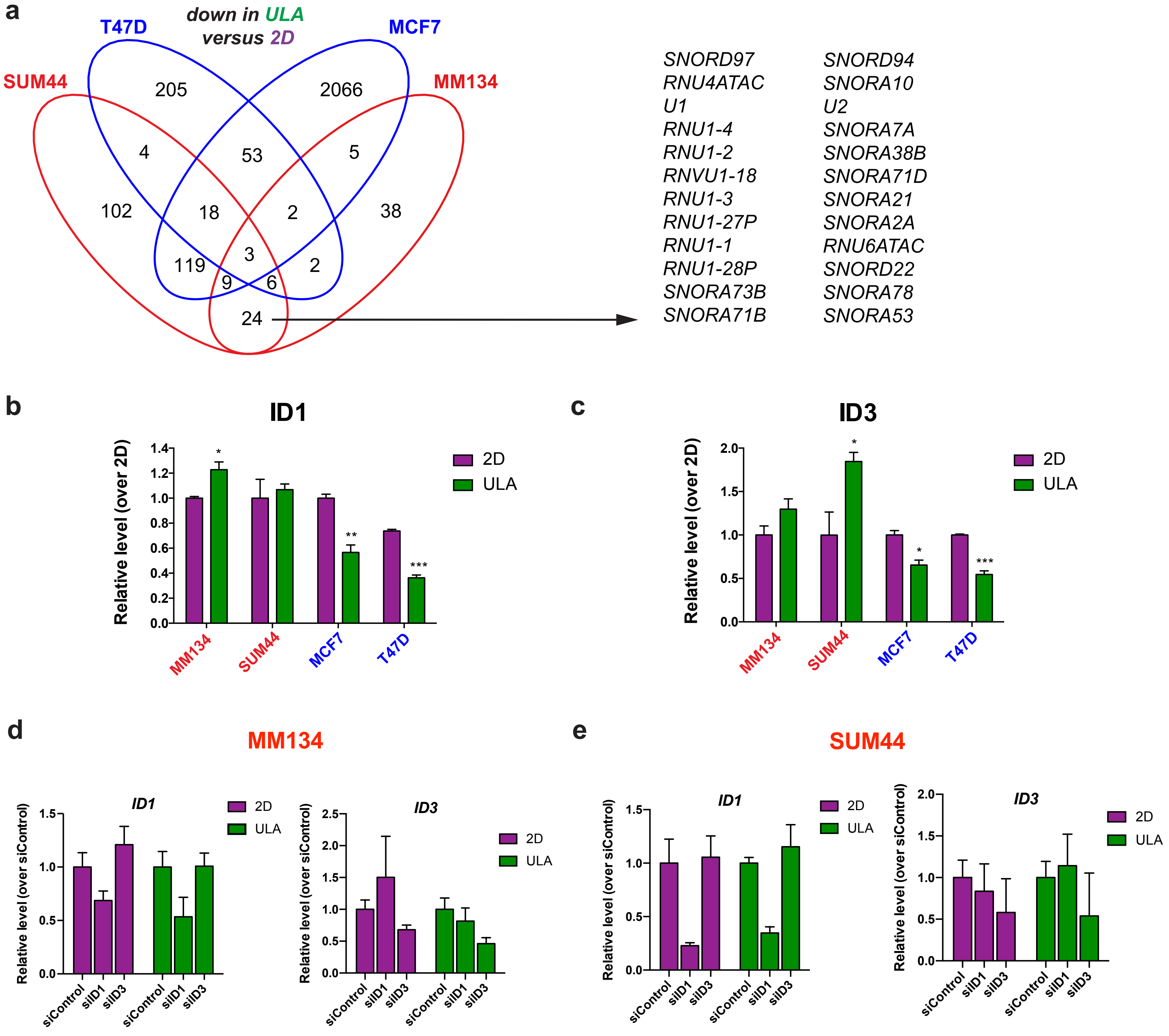

Supplementary Figure S9. Transcriptomic profiling of ILC and IDC cell lines in 2D and ULA culture. (**a**) Venn diagrams showing the overlap between the genes downregulated after 24 hours in ULA (green) culture as compared to 2D (purple) in ILC (red) and IDC (blue) cell lines. The list on the right shows the 24 genes commonly downregulated in the two ILC but not the IDC cell lines, mostly made up of small nuclear, nucleolar and spliceosomal RNAs. (**b-c**) Quantification of ID1 (**b**) and ID3 (**c**) immunoblots from **Figure 5d**. Data is displayed as mean +/- standard error relative to the 2D condition in each cell line. Graphs show data from three biological replicates. p-values (**d-e**) qRT-PCR validation of the *ID1* (left) and *ID3* (right) knockdown in MM134 (**d**) and SUM44 (**e**) cells 4 days after transient transfection with the indicated siRNAs. Data is displayed as mean +/- error relative to siControl in each condition in each cell line. p-values are from t-tests between 2D and ULA for each siRNA. * p ≤ 0.05, ** p ≤ 0.01; *** p ≤ 0.001.

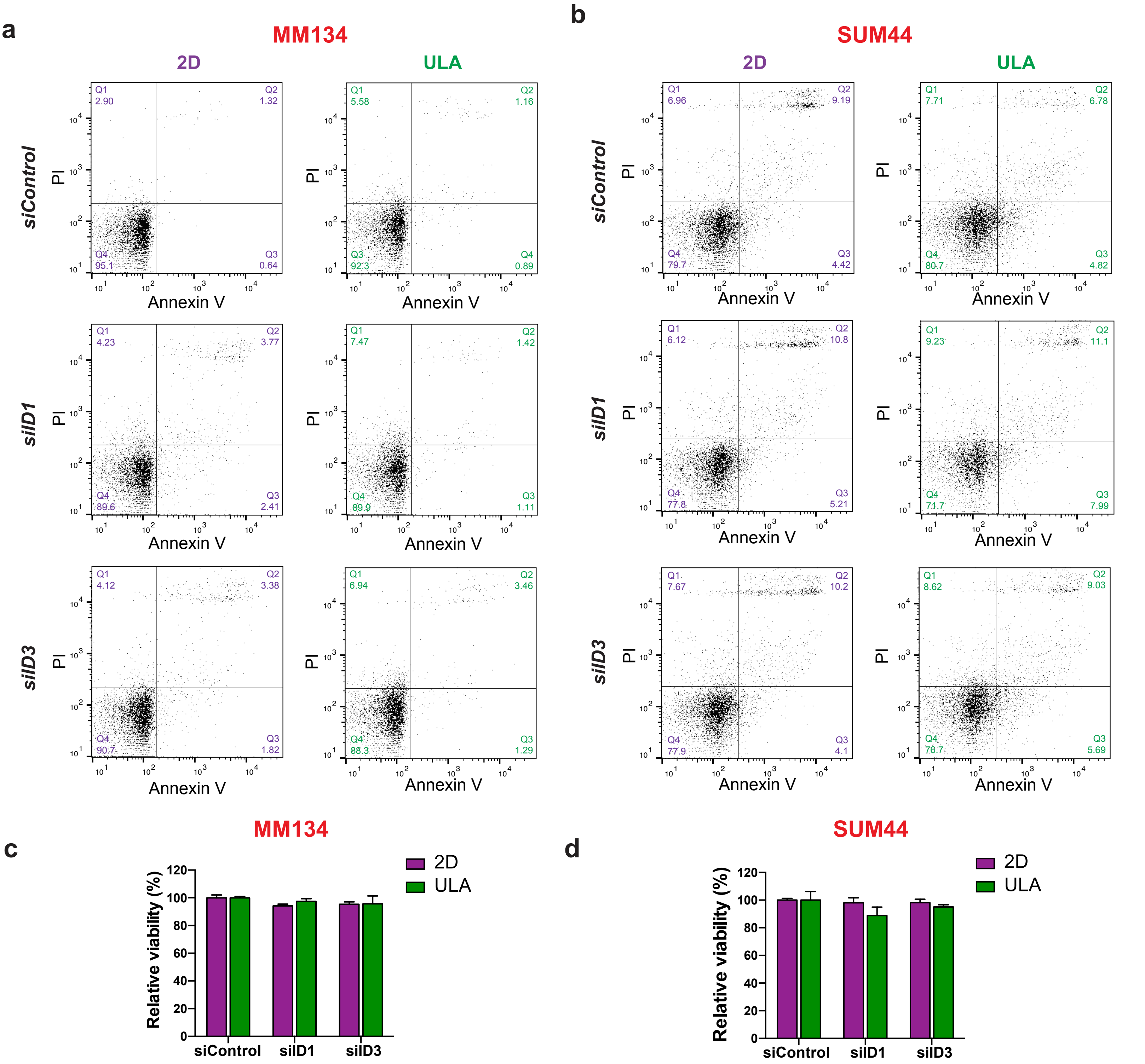

Supplementary Figure S10. Anoikis resistance in ILC cell lines with *ID1* or *ID3* knockdown in 2D and ULA culture. (**a-b**) Representative Annexin V and PI FACS staining plots of the of the ILC cell lines (**a**) MM134 and (**b**) SUM44 6 days after transient transfection with the indicated siRNAs in 2D (left; purple) or ULA (right; green) culture. (**c-d**) Quantification of the viable (Q4: Annexin V-/PI-) population in (**c**) MM134 and (**d**) SUM44 ILC cell lines from (**a-b**). Data is displayed as mean percentage +/- standard deviation relative to siControl in each condition in each cell line. Graphs show representative data from two experiments (n=3).

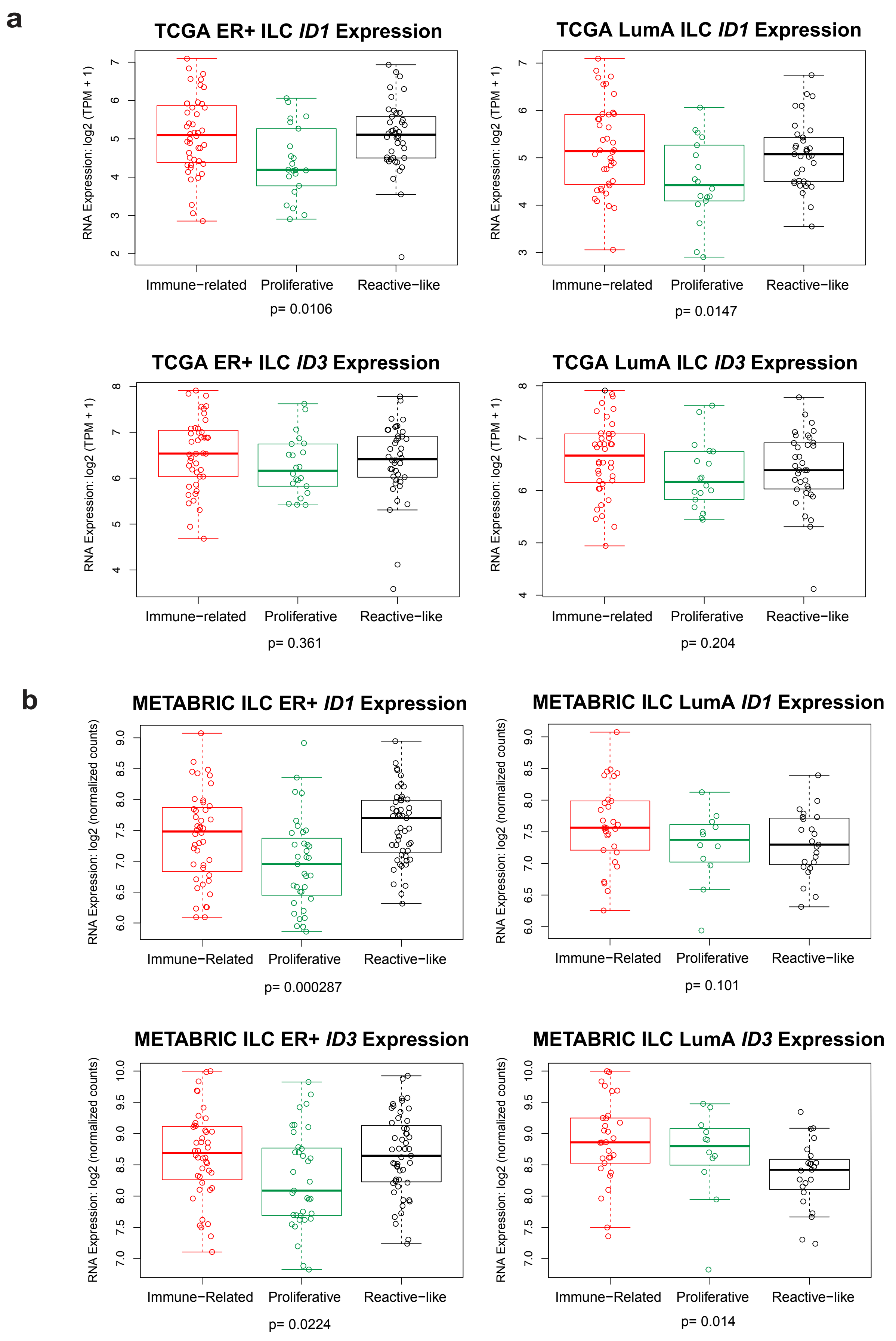

Supplementary Figure S11. *ID1* and *ID3* expression in the molecular subtypes of ILC. (**a-b**) mRNA levels of *ID1* (top) and *ID3* (bottom) in ER-positive (left) and LumA (right) of ILC tumors from the (**a**) TCGA and (**b**) METABRIC cohorts according to the the immune-related (red), proliferative (green) and reactive-like (black) molecular subtypes. p-values are from on-way ANOVA with Benjamini-Hochberg multiple comparison test.

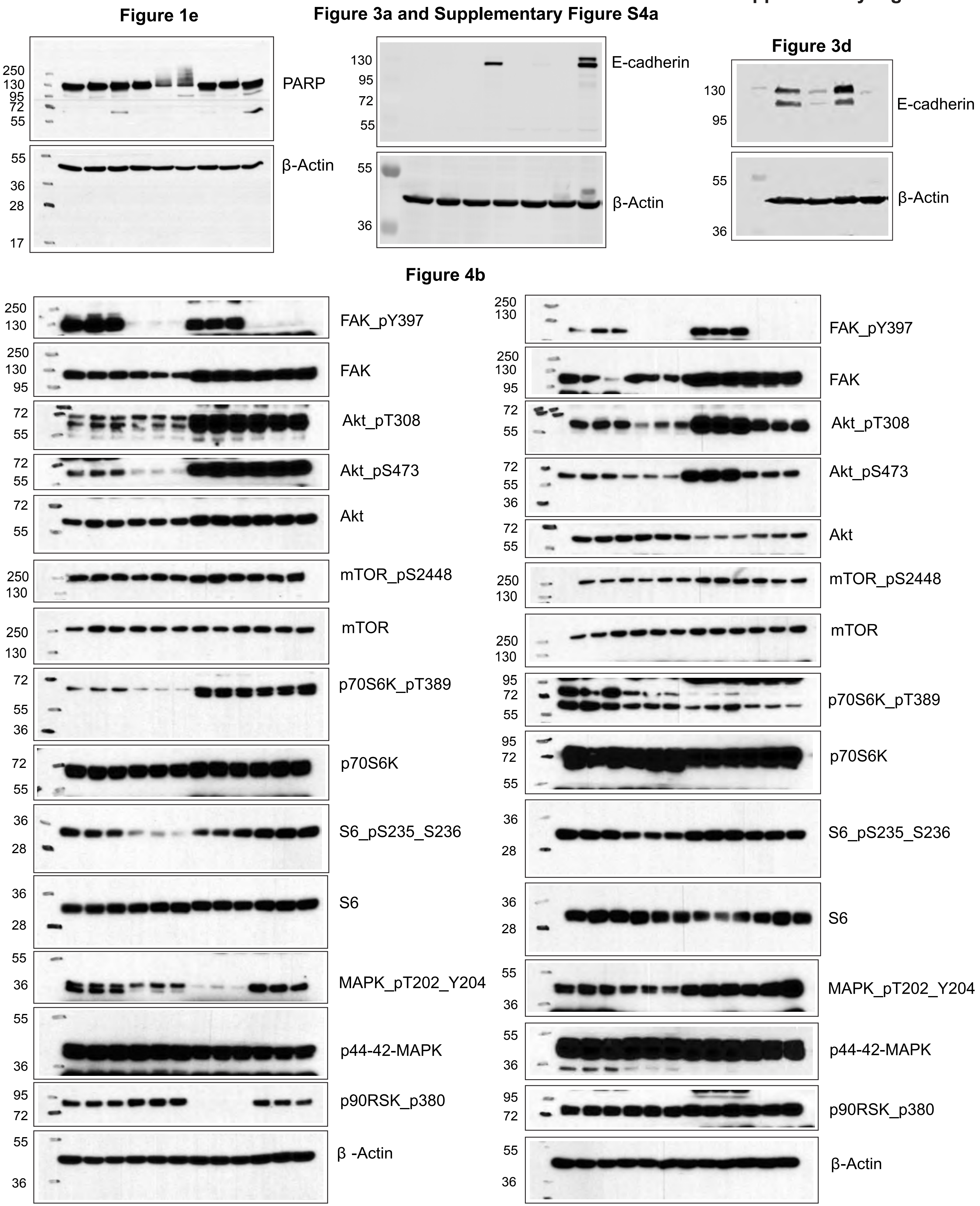

Supplementary Figure S12. Uncropped blots related to Figures 1e, 3a, 3d, 4b and Supplementary Figure S4a.

Figure 5d

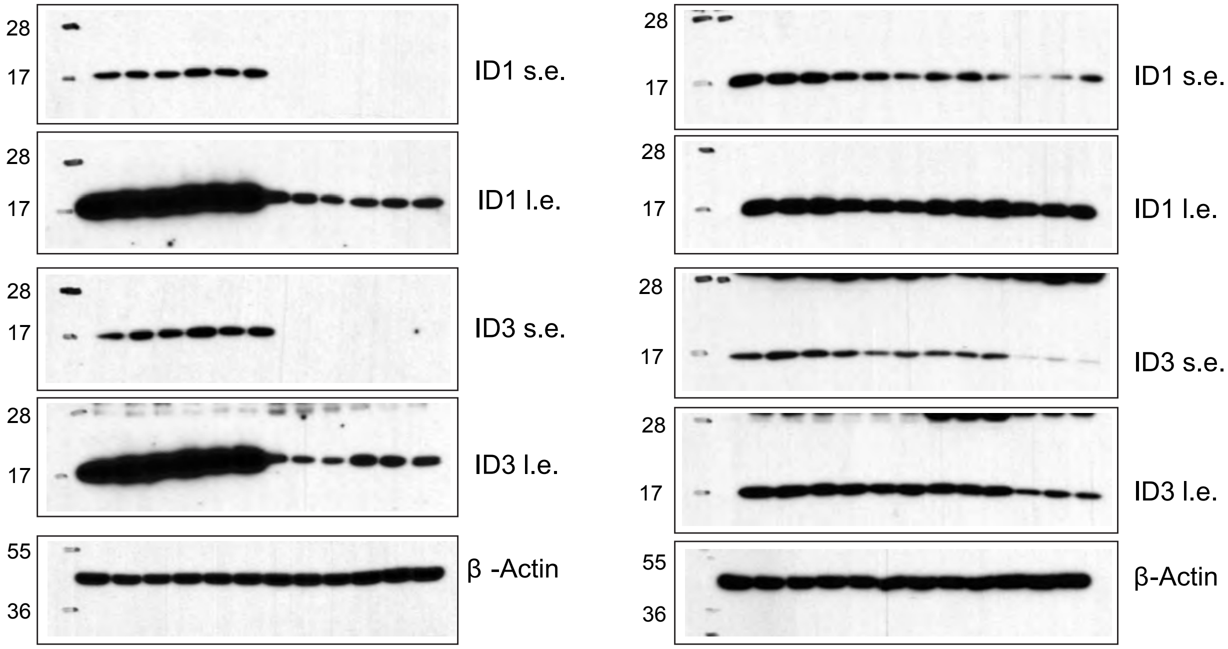

Figure 5e

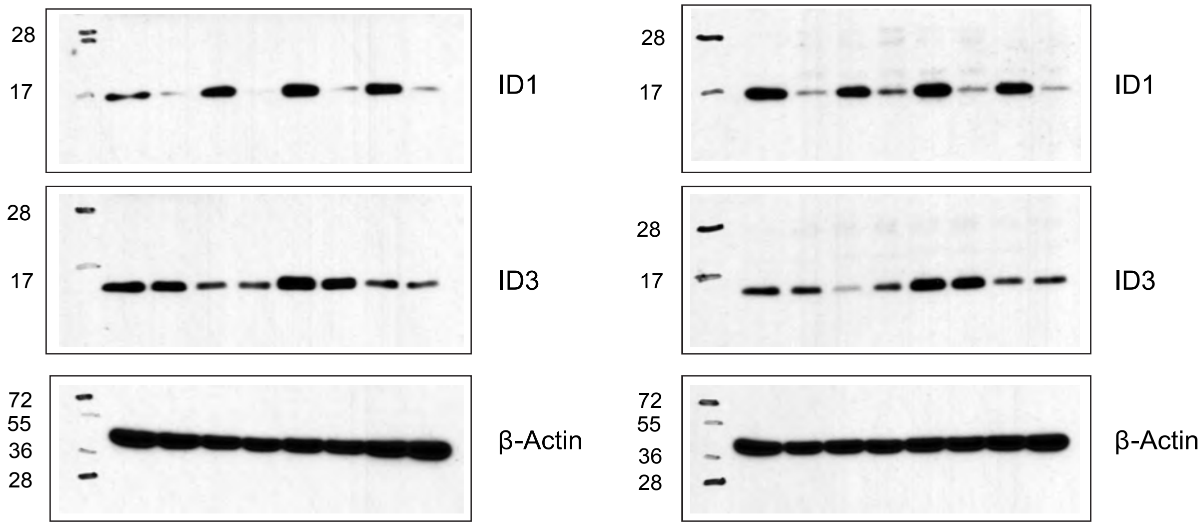

Supplementary Figure S13. Uncropped blots related to Figures 5d and 5e. l.e.: long exposure. s.e.: short exposure.

Supplementary Figure S14

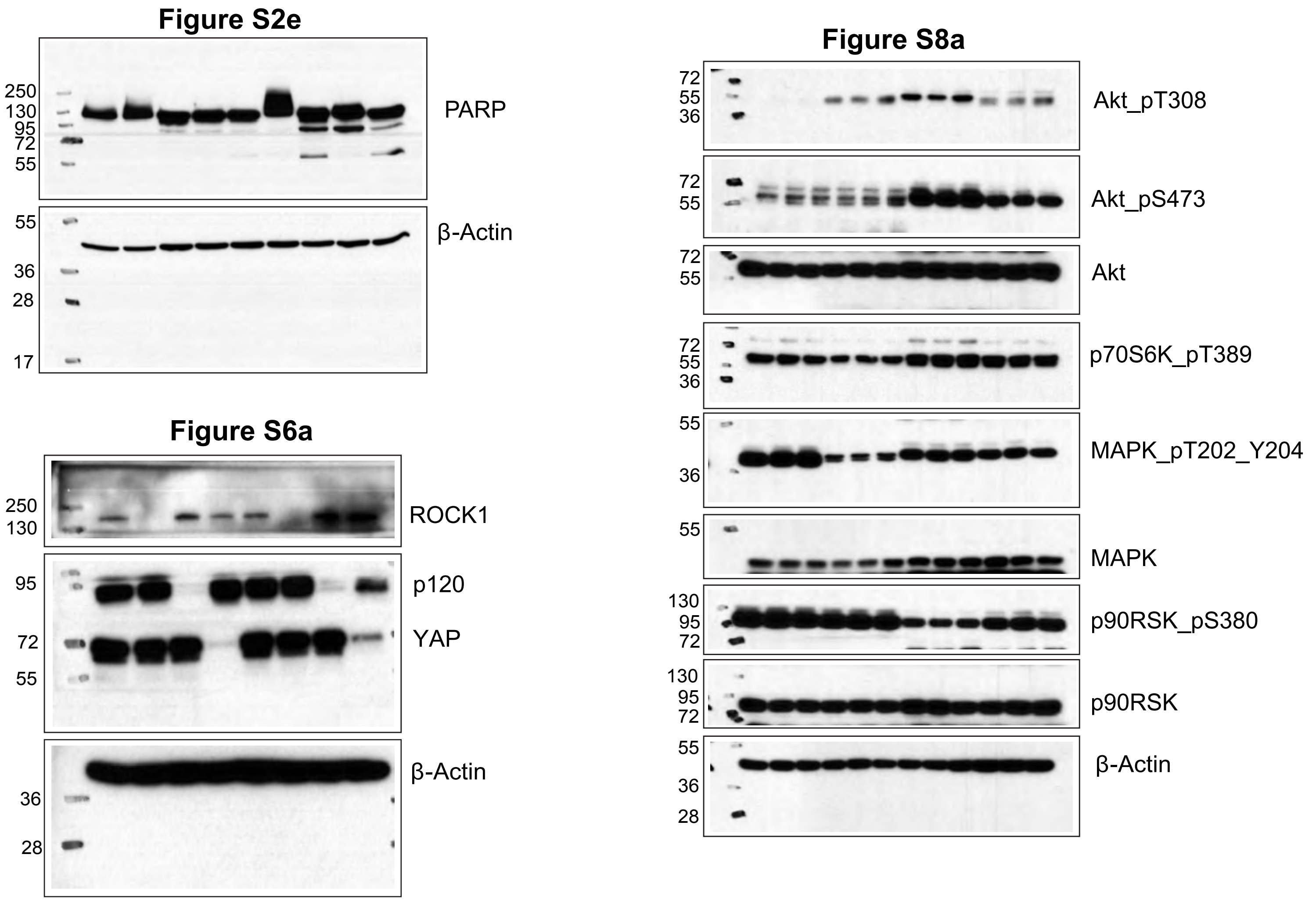

Supplementary Figure S14. Uncropped blots related to Supplementary Figures S2e, S6a and S8a.
